# *Cryptosporidium* oocyst wall proteins are true components of the oocyst wall and *COWP8* is not required for parasite transmission

**DOI:** 10.1371/journal.ppat.1013561

**Published:** 2025-10-23

**Authors:** Ross Bacchetti, Sarah Stevens, Leandro Lemgruber, Mariana Azevedo Gonzalez Oliva, Emma M. Sands, Konstantinos Alexandrou, Michele Tinti, Lee Robinson, Jack C. Hanna, Simona Seizova, Peyton Goddard, Massimo Vassalli, Mattie Christine Pawlowic

**Affiliations:** 1 Wellcome Centre for Anti-Infectives Research, School of Life Sciences, University of Dundee, Dundee, United Kingdom; 2 Cellular Analysis Facility, MVLS-Shared Research Facilities, College of Medical, Veterinary & Life Sciences, University of Glasgow, Glasgow, United Kingdom; 3 James Watt School of Engineering, University of Glasgow, Glasgow, United Kingdom; Boston Children's Hospital, UNITED STATES OF AMERICA

## Abstract

Cryptosporidiosis is a significant cause of diarrhoeal disease contributing to substantial morbidity and mortality for the immunocompromised and for young children, especially those who are malnourished. There are no vaccines and no effective treatments for these patients. *Cryptosporidium* parasites are transmitted as an oocyst, which is composed of a hardy oocyst wall that protects parasites in the environment. Oocysts are often waterborne, and are resistant to common water treatments, including chlorination. Little is understood about how the *Cryptosporidium* oocyst is constructed, its composition, and the how it resists chlorination. A family of nine *Cryptosporidium* Oocyst Wall Proteins (COWPs) is predicted from the genome. However, due to the technical challenges of working with this parasite in the laboratory, only *cowp1* has been investigated to date. Using CRISPR/Cas9, fluorescent fusions were generated for the remaining members of the family, *COWP*s 2–9. Microscopy confirms that all *COWP*s localise to the oocyst wall. Further, *COWP*s 2–4 appear to localise specifically to the oocyst suture, the site from which parasites emerge from the oocyst during infection. *Cowp* 6 and 8 were observed to be expressed by female parasites. These proteins localise to puncta consistent with organelles called wall forming bodies. These organelles store and then secrete material to form the oocyst wall. Parasites lacking *cowp8* produce viable oocysts that have typical oocyst morphology. *Cowp8* knockout oocysts are transmissible under laboratory settings and readily infect immunocompromised mice. Biomechanical measurements determine that *COWP8* is not required for the strength of the oocyst wall. This work confirms the role of *cowp*s in oocyst wall formation and sets a foundation for further exploration of the role of these proteins in transmission of *Cryptosporidium* parasites.

## Author summary

Infection with *Cryptosporidium* causes diarrhoea, which can be fatal for malnourished children or people who are immunocompromised. There is no vaccine or effective medicines available to treat these patients. Infection with *Cryptosporidium* typically starts with drinking contaminated water that contains the parasites. Parasites are transmitted as an “oocyst” that has a hardy shell that protects parasites. This shell makes the parasites resistant to chlorination, the most common method of water disinfection. How these parasites construct this protective shell and how the shell is so resilient is unknown. When the genome of *Cryptosporidium* was sequenced, a group of nine proteins were predicted as shell proteins. Only one protein from this group has been studied to date. We use genetics and microscopy to confirm that the other eight proteins are involved in constructing the parasite shell. Three of these proteins appear to be specific to the seam on the shell that opens after parasites are ingested. Surprisingly, we were able to completely remove one of these genes from the genome*, cowp8.* Parasites that lack *cowp8* are still able to construct normal shaped shells and are infectious. Using biomechanics, we find that the shells are just as strong whether they have the *COWP8* protein or not. This forms a strong foundation of tools and insight to guide further investigation of the role of the shell in parasite transmission.

## Introduction

Diarrhoeal disease is a major cause of morbidity and mortality for children under five years old, especially in low- and middle-income countries. The Global Enteric Multicentre Study revealed that cryptosporidiosis is the second leading cause of moderate to severe diarrhoeal disease [1]. Cryptosporidiosis was estimated to cause 8.2 million disability adjusted life years (DALYs) and 133,422 deaths in 2019 [2]. Recurrent diarrhoeal episodes associated with cryptosporidiosis leads to alteration of gut morphology, growth stunting, delayed development, and impaired cognitive function [3]. There are no vaccines available, and the only drug approved to treat cryptosporidiosis is not effective in the populations that need it most—malnourished children and immunocompromised adults [4].

Another major challenge of combatting this disease is the difficulty in removing parasites from the environment. *Cryptosporidium* is transmitted as an oocyst, an exceptionally hardy structure, granting protection to the four internal sporozoites. *Cryptosporidium* is a faecal-oral pathogen, and infection occurs by ingestion of contaminated food or water. *Cryptosporidium* oocysts are resistant to most water treatments including chlorination [5]. The ability to resist chemical disinfection contributes to cryptosporidiosis as the leading cause of reported waterborne outbreaks in high income countries [6], which are currently on the rise in the UK [7]. Outbreaks are common in recreational facilities that depend on chlorination for water treatment, daycare centres that care for young children, and in communities after large water treatment facilities fail, often due to increased influx of rainwater. Some species of *Cryptosporidium* also infect cattle and wildlife and can cause zoonotic infections [8]. When waterborne outbreaks occur, especially those in close proximity to land used for agriculture, cattle are often blamed as the source of the oocysts that have contaminated the water. More work is required to understand the link between animal, human, and environmental health.

*Cryptosporidium* oocysts are slightly ovoid in shape and are approximately five microns in diameter [9]. The oocyst wall is made of three components: the outer oocyst wall, the inner oocyst wall, and the suture. The outer wall is SDS- and protease-resistant, made of acid-fast lipids, similar to the outer membrane of mycobacteria, and is sensitive to organic solvent treatment [10,11]. This acid-fast lipid outer layer is hypothesised to act as a waxy coating that aids environmental survival, particularly protection from disinfectants and chlorination. The identity of the acid-fast lipids is still to be determined. The inner layer of the *Cryptosporidium* oocyst is composed of fibrillar glycoproteins. Once the oocyst wall is opened, the inner layer is susceptible to degradation by proteases [10]. The protein constituents of the inner wall are poorly characterised but appear to be highly crosslinked [11,12]. It is hypothesised that these proteins offer structural strength and rigidity to the oocyst. The oocyst suture is a predefined opening, which appears to span approximately one-third to one-half the oocyst’s circumference, acting as the exit point for sporozoites [13]. Electron micrographs of the suture in cross-section reveal that the structure is formed from “zipper-like” teeth on the inside of the oocyst wall [14]. After the oocyst has been ingested, the suture opens allowing sporozoites to escape and invade the gut [15,16]. The triggers for oocyst excystation and suture opening are unknown. However, the presence of enzymes and bile salts in the intestinal lumen, changes in CO_2_ concentration, changes in temperature, or the onset of reducing conditions have been implicated [17]. Excystation can be triggered in the laboratory by simply incubating oocysts at 37 °C.

There are few experimentally validated molecular markers for the outer and inner oocyst walls and there are no markers for the suture. The *Cryptosporidium* genome contains a family of nine predicted *Cryptosporidium* oocyst wall proteins, or *cowp*s [18,19]. Recent proteomics analysis using hyperplexed localization of organelle proteins by isotope tagging (hyperLOPIT) identifies additional potential oocyst wall proteins beyond the *COWP*s [20]. All *COWP*s have a signal peptide, lack transmembrane domains, and are rich in cysteines. These cysteines occur at a high frequency whereby every 10^th^-12^th^ amino acid is a cysteine residue. The abundance of cysteines in the *COWP*s suggest the possibility to form inter- and intramolecular disulphide bonds, providing stability to the oocyst wall [21]. *Cowp1* is experimentally confirmed to be expressed by female parasites in wall forming body organelles (WFB) and then secreted to form the oocyst wall [22,23]. With the advent of CRISPR, reporter parasites confirmed that female parasite stages are present in *in vitro* culture and that *COWP1* is one of the first proteins expressed by female parasites. Additionally, *COWP1* was localised in female parasites to puncta consistent with WFBs, and in purified oocysts was localised to the oocyst wall. Antibodies generated against *COWP8* suggest that this protein is also an integral inner wall protein [19]. The remaining *COWP*s have remained hypothetical oocyst wall proteins and their function in parasite transmission is unexplored. Here, we determine that all members of the *COWP* family are true oocyst wall proteins. This includes three proteins that localise to the suture, providing the first markers for this important structure. We investigated the function of the highly abundant oocyst wall protein, *COWP8*, in oocyst wall structure and parasite transmission.

## Results

### *COWP*s localise to the oocyst wall and suture

**Fig 1:**
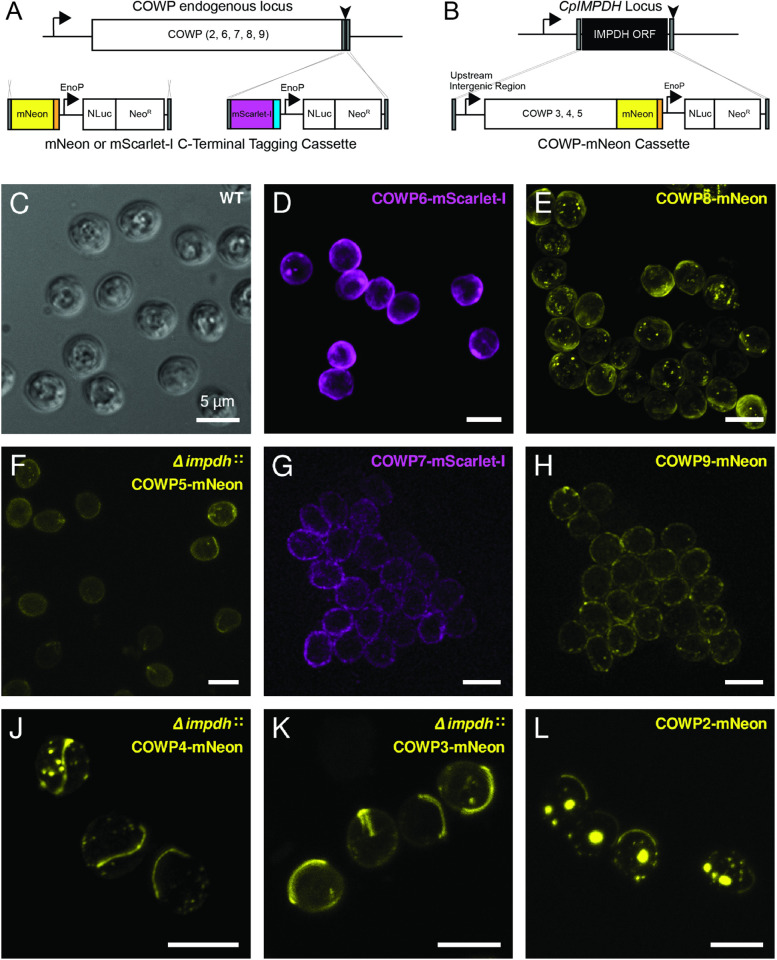
*Cryptosporidium* Oocyst Wall Protein (COWP) family are confirmed oocyst wall proteins. A) CRISPR/Cas9 was used to produce strains of *Cryptosporidium* where *COWP2*, *6-9* are individually fused at their C-terminus to a fluorescent protein, either mNeon (yellow) or mScarlet-I (purple). B) Expression of *COWP3-5* tagged at the C-terminus with mNeon were driven by their native promoter and targeted for integration at the *Cpimpdh* locus. C) Live microscopy of wild type *Cryptosporidium parvum* oocysts. D-L) Fluorescence microscopy of live oocysts confirms that *COWP*s localise to the oocyst wall and/or suture. Images collected, as indicated, using a Deltavision widefield epifluorescence microscope, or on a Zeiss LSM880 Airyscan microscope either in confocal or super resolution mode (Airyscan mode). Representative images shown; contrast adjusted individually for each image and not normalised. Scale bar for all images is 5 µm. Image collection parameters for each image: C) widefield epifluorescence microscope, single z-plane, exposure time 0.08 msec, laser 50%. D) confocal, projection of 29 z-planes, exposure time 0.01 msec, laser power 2%. E) super resolution, projection of 20 z-planes, exposure time 0.038 msec, laser power 2.2%. F) confocal, projection of 13 x-planes, exposure time 0.02msec, laser power 2% G) widefield epifluorescence microscope, single z-plane, exposure time 0.5 sec, laser 50%. H) widefield epifluorescence microscope, projection of 12 z-planes, exposure time 0.5 sec, laser 50%. J) super resolution, 39 z-planes, exposure time 0.043 msec, laser power 2.3%. K) confocal, projection of 10 z-planes, exposure time 0.02 msec, laser power 2%. L) confocal, projection of 16 z-planes, exposure time 0.02 msec, laser power 2%.

To determine if *COWP2–9* are oocyst wall proteins, CRISPR/Cas9 was used to generate reporter strains. Strains were engineered to have both a fluorescent protein and epitope tag, either mNeon-3 × HA or mScarlet-I-3 × myc (Fig 1A). Because *COWP*s contain an N-terminal signal peptide sequence, strains were designed as C-terminal fusions. Reporter strains were successfully generated for all *cowp*s (S1–S4 Figs). We were unable to recover mutants where *COWP3–5* were tagged at the C-terminus, therefore an alternative tagging strategy was employed where a second copy of these *cowp*s was introduced at a non-essential locus (Fig 1B). The corresponding promoters (entire upstream intergenic region) and open reading frame for *cowp3–5* were cloned into the tagging constructs (mNeon-3 × HA). CRISPR was used to insert these expression cassettes at *Cpimpdh* (S2 Fig)*.*

Oocysts were purified from faecal samples and examined live by fluorescence microscopy (Fig 1C). *COWP5–9* localise across the oocyst wall (Fig 1D–H) in a similar pattern as previously observed for *COWP1* [23]. Additionally, immunoelectron microscopy confirmed localisation of *COWP6* and *COWP8* to the oocyst wall (S5 Fig). Fluorescence microscopy indicates that *COWP8* oocysts contain a few very small puncta in the space between sporozoites and the oocyst wall that are not associated with the residual body (Fig 1E). These were also observed by immunoelectron microscopy as globules of wall material secreted by the zygote that did not incorporate into the inner oocyst wall (S5C Fig). Fluorescent signals for *COWP5*, *COWP7* and *COWP9* were observed to be of lower intensity relative to *COWP6* and *COWP8* (Fig 1F–H). The expression level apparent in these images are consistent with published transcriptomics data indicating that *cowp1*, *cowp6*, and *cowp8* are the most abundant members of this family (S6 Fig) [23,24]. As the fluorescence signal of COWP5-mNeon was particularly dim, a second reporter strain was generated where the promoter of *cowp1* is used to drive expression of *cowp5* (S7A–C Fig). Live fluorescence microscopy further confirms that *COWP5* is localised to the oocyst wall (S7D Fig).

Notably, *COWP2–4* localise to the oocyst wall, but more specifically their localisation pattern is consistent with the suture, in both shape and length. *COWP4* is located at the suture and in small puncta, similar to the structures observed in the *COWP8* strain (Fig 1J). Unlike the other *COWP*s that localise evenly across the oocyst wall (*COWP*s 1,5,6,7,8,9), *COWP3* localises across the oocyst wall but is highly concentrated at the suture (Fig 1K). COWP2-mNeon localises to the suture, but also to a focus corresponding to the residual body (Fig 1L).

To test whether localisation of COWP2-mNeon to the residual body was a mislocalisation due to fusion with the mNeon protein, a second transgenic strain was generated where *COWP2* is fused to the epitope tag alone, COWP2-HA (S1A Fig). COWP2-mNeon and COWP2-HA oocysts were excysted, labelled with anti-HA antibody and inner oocyst wall antibody (1E3; Kerafast) and imaged. In excysted oocysts of both tagged strains, *COWP2* is localised in the residual body and present at the suture (S8 Fig). This confirms that *COWP2* localisation is unlikely to be an artefact of the bulky tag but truly localises to the residual body. Further, COWP2-HA was only detected in excysted oocysts, suggesting that *COWP2* is an inner wall protein. The relatively low protein abundance of the other *COWP*s prevented observation of labelling by immunoelectron microscopy.

Application of genetics and microscopy confirms that all members of the *cowp* family are true oocyst wall proteins. Further, we discovered that *COWP*s 2–4 specifically localise to the *Cryptosporidium* oocyst suture, providing the first markers for this structure.

### *COWP6* and *COWP8* are expressed by macrogamonts exclusively and are secreted after fertilisation to form the oocyst wall

To determine which parasite life cycle stages express *cowp*s, reporter strains were cultured *in vitro* or *in vivo* and expression of *cowp*s was observed using fluorescence microscopy. We focussed on *COWP6* and *COWP8* as expression was high enough to confidently observe by fluorescence microscopy. *COWP5* was not attempted; *COWP7* and *COWP9* were examined, but expression could not be visualised by fluorescence microscopy, likely due to low expression levels.

We lack markers for discrete life cycle stages and use shape and number of nuclei to differentiate life cycle stages. Expression of *COWP6* and *COWP8* was undetectable in asexual stages including single nuclei trophozoites (S9A Fig) and eight-nuclei meronts (S9B Fig). Males (microgamonts), which have up to 16 bullet-shaped nuclei, also do not express *COWP6* or *COWP8* (S9C Fig).

**Fig 2:**
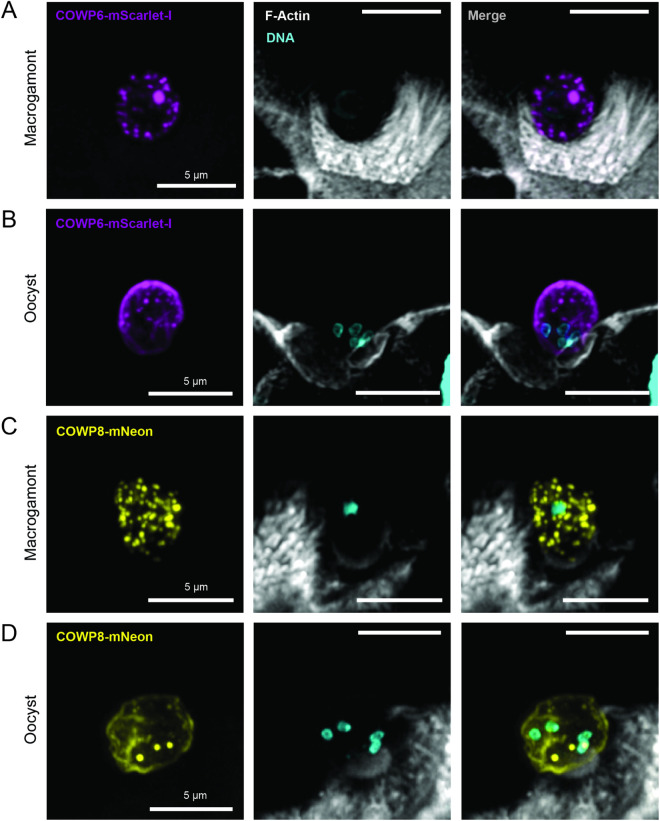
*COWP*s are expressed by macrogamonts and are secreted to form the oocyst wall. Super resolution microscopy of ileal tissue collected from IFN-γ knockout mouse infected with COWP6-mScarlet-I (A and B) or COWP8-mNeon (C and D) strains. Mice were culled at peak infection and tissue was processed for histology. Macrogamonts are identified by size (~ 5 µm) and a single nucleus; oocysts are identified similarly by size and presence of 4 nuclei. Brush border (F-actin stained with phalloidin-AlexaFluor-647, white) and nuclei (DNA stained with Hoechst, cyan). A) Prior to fertilisation, *COWP6* (purple) is localised in numerous puncta in the female parasites, resembling wall forming body organelles. B) After fertilization, *COWP6* is secreted to form the oocyst wall. C) *COWP8* (yellow) is localised in puncta similar in size and number to *COWP6*, and D) after fertilisation is secreted to form the oocyst wall. Small puncta of COWP8-mNeon remain after oocyst wall formation, consistent with live microscopy of oocyst purified from faecal material (reported in Fig 1E). Images collected on a Zeiss LSM880 Airyscan microscope, Airyscan mode. Representative images shown; contrast adjusted individually for each image and not normalised. Scale bar for all images is 5 µm. Image collection parameters for each image: A) projection of 5 z-planes, exposure time mScarlet-I 0.028 msec and laser 2%, exposure time Alexa-647 0.024 msec and laser 0.5%, exposure time Hoescht 0.010 msec and laser 3%. B) projection of 10 z-planes, exposure time mScarlet-I 0.028 msec and laser 2%, exposure time Alexa-647 0.024 msec and laser 0.5%, exposure time Hoescht 0.010 msec and laser 3%.C) projection of 9 z-planes, exposure time mNeon 0.023 msec and laser 2.4%, exposure time Alexa-647 0.020 msec and laser 0.5%, exposure time Hoescht 0.022 msec and laser 3%. D) projection of 17 z-planes, exposure time mNeon 0.020 msec and laser 2.4%, exposure time Alexa-647 0.020 msec and laser 0.5%, exposure time Hoescht 0.022 msec and laser 3%.

*COWP6* and *COWP8* expression was exclusively observed in females, called macrogamonts (Fig 2A and 2C). Macrogamonts have a single nucleus and synthesise the components of the oocyst wall as they mature [23]. These components are stored in organelles called “wall forming bodies” (WFB) which have a punctate pattern [22]. Previously, *COWP1* was localised to wall forming bodies by immunoelectron microscopy [22]. Recent examination of COWP1-mNeon parasites identified a similar punctate localisation consistent in size, shape, and number with WFBs [23]. *COWP6* and *COWP8* reporter strains produce the same WFB-like localization pattern. Prior to fertilisation (characterised by a single large nuclei), macrogamonts parasites express *COWP6* (Fig 2A) and *COWP8* (Fig 2C) in numerous puncta of a similar, and small size. After fertilisation and meiosis (four nuclei observed) *COWP*s are secreted to form the oocyst wall (Fig 2B and 2D).

### *Cowp*8 is not essential for parasite survival, infection, or transmission

**Fig 3:**
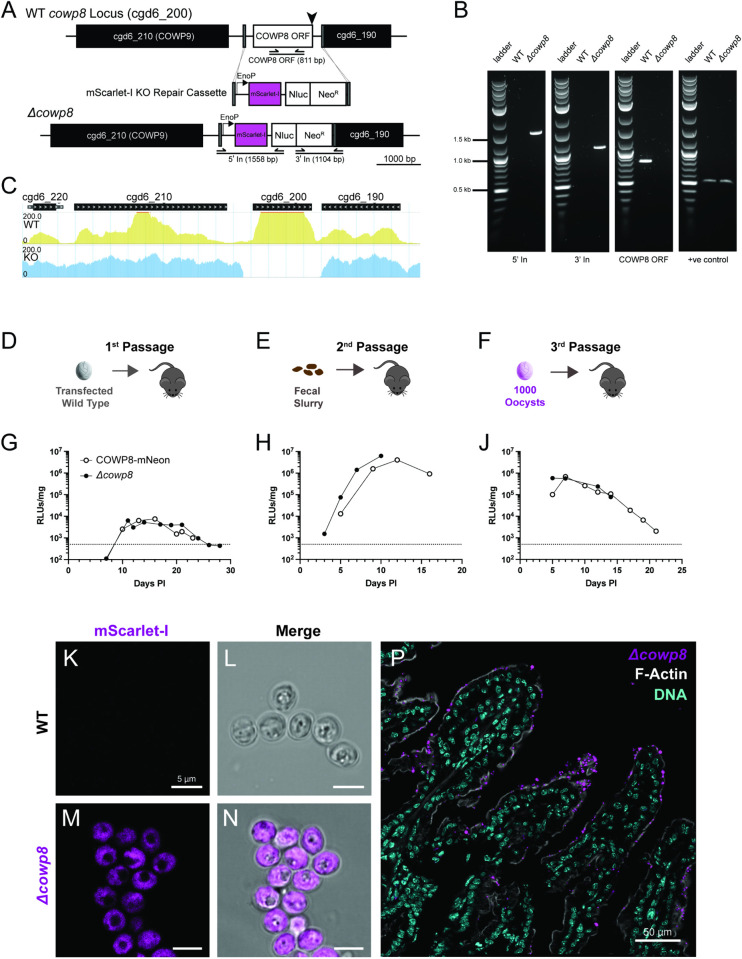
*COWP8* is not essential for infection or transmission. A) Strategy to replace the full open reading frame of *cowp8* (cgd6_200) with a repair cassette where mScarlet-I and NanoLuciferase-Neomycin resistance fusion protein (NLuc-Neo^R^) is expressed by the constitutive *Enolase* promoter. The same gRNA (black arrow) that was used to target the C-terminus for fluorescent fusion and the same downstream region of 50 bp of homology (grey) was used to target the gene for deletion (S4A Fig). B) PCR with primer pairs indicated in (A) was performed using genomic DNA extracted from wild type and *∆cowp8.* C) Whole genome sequencing of wild type (yellow) and *∆cowp8* (blue) confirms gene deletion. Histogram illustrates sequencing coverage mapped at the *cowp8* locus. Confocal microscopy of live wild type (D and E) and *∆cowp8* (F and G) confirms mScarlet-I cytoplasmic expression of *∆cowp8* strain (magenta); scale bar 5 µm. H) Super resolution microscopy of tissue collected from interferon-gamma knockout mouse infected with *∆cowp8* culled at peak infection and processed for histology. Brush border (F-actin stained with phalloidin, white) of ileal villi (DNA stained with Hoechst, cyan) are highly infected with *∆cowp8* (mScarlet-I expressed in the parasite cytoplasm, purple). Images collected on a Zeiss LSM880 Airyscan microscope, (Airyscan mode for P). Representative images shown. J-L) Experimental design for passage of *∆cowp8* strain in interferon-gamma knockout mice. J) Initial passage where inoculum is wild type sporozoites transfected with CRISPR machinery to target *co*wp8** for deletion, K) second passage infected with slurry made of faecal material from first passage, L) and third passage infected with oocysts purified from the second passage. Infection level of COWP8-mNeon (white circles) and *∆cowp8* strains (black circles) is similar and follows typical acute infection pattern in M) first, N) second, and P) third passages. Infection level of mice as determined by faecal NLuc, limit of detection at 500 RLU/mg, dotted line. Average and SD of three technical replicates of one biological replicate.

*Cowp8* is a highly expressed member of the *cowp* family, at levels similar to *cowp1*. From transcriptomics data, *cowp8* is in the 99% percentile of genes expressed by macrogamonts and is slightly higher in expression than *cowp1* (bulk RNASeq from female parasites; S6 Fig) and is the last *cowp* to be expressed during female maturation (single cell RNASeq) [23,24]. When measured by proteomics, abundance of *COWP8* is only slightly lower than *COWP1*. To understand the role of *cowp*s in construction of the oocyst wall, infection, and parasite transmission, we used CRISPR/Cas9 to generate parasites lacking *cowp8*. The repair cassette was designed to replace *cowp8* (cgd6_200) with an mScarlet-I fluorescent protein and the NLuc-Neo^R^ fusion protein, driven by a strong, constitutive promoter (Fig 3A).

Diagnostic PCR and whole genome sequencing confirmed that parasites lacking *cowp8* were successfully generated (Fig 3B and 3C). Using confocal microscopy *Δ*cowp8** oocysts display no difference in morphology from wild type (Fig 3K–N). Parasite burden of*Δ*cowp8**-infected tissue was observed by fluorescence microscopy. Intestinal tissue was collected from mice at peak infection levels (determined by faecal NLuc) and imaged. High levels of infection were observed along the length of the villi (Fig 3P). *Δ*cowp8** parasites were viable *in vivo* (Fig 3D–J) and parasite shedding followed a typical acute pattern, indistinguishable from the pattern observed for passage of the COWP8-mNeon reporter strain (Fig 3G and 3J). Infection was unaffected by type of inoculum; oral gavage with either a faecal sample (Fig 3E) or purified oocysts (Fig 3F) both generated robust infection.

Although *cowp8* is not required for infection of immunocompromised mice under laboratory conditions, it could be required for survival in the environment. Oocysts are resistant to treatment with chlorination and can survive exposure to high levels of bleach. *∆cowp8* oocysts were assessed for their ability to survive treatment with bleach. COWP8-mNeon and *∆cowp8* oocysts were treated with 8% bleach, washed thoroughly, and used to infect mice. No difference in infection levels was observed for *∆cowp8* parasites (S10A Fig and S10B), confirming that oocysts remain resistant to chlorination despite loss of *cowp8*. In addition to their resistance to disinfectants, *Cryptosporidium* oocysts can persist and survive for considerable lengths of time. Oocysts stored below 15 °C remain infectious even after six months [25]. Parasite infectivity begins to be greatly compromised only when oocysts are stored at temperatures above 37 °C [5,26]. We tested the effect of heat treatment on oocysts lacking *cowp8*. Treatment at 60 °C for 5 minutes was sufficient to render both COWP8-mNeon and *∆cowp8* non-infectious (S10A Fig and S10C).

### Loss of *cowp8* does not alter oocyst morphology

**Fig 4:**
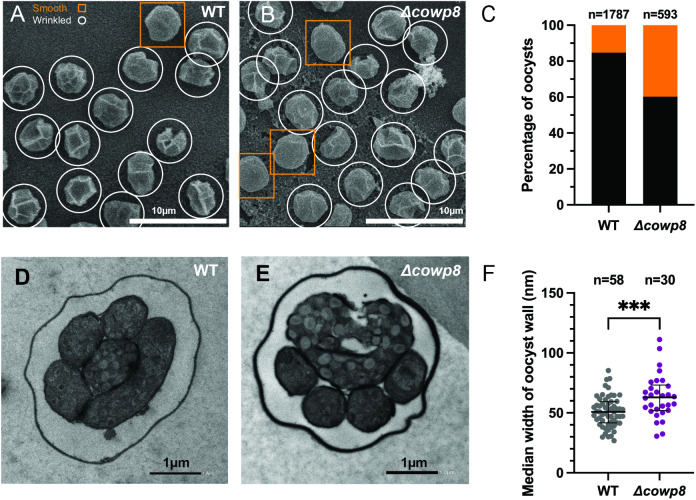
*COWP8* does not alter robustness of oocyst wall. Representative field of view of A) WT and B) *Δcowp8* oocysts imaged using Scanning Electron Microscopy (SEM). ‘Wrinkled’ oocysts are circled in white and ‘Smooth’ oocysts are outlined with orange squares. Scale bars are 10 µm. C) Percentage of oocysts that appear wrinkled (black) or smooth (orange) quantified. *n* = 1787 WT oocysts (1514 wrinkly (84.7%) and 273 smooth (15.3%)). *n* = 593 for *Δ*cowp8** oocysts (357 wrinkly (60.2%) and 236 smooth (39.8%)). Raw data in S5 Table. Representative Transmission Electron Microscopy was performed on D) wild type and E) *Δcowp8* oocysts. Scale bars are equivalent to 1µm. F) Median width of oocyst wall. Error bars, IQR; ***p *<* 0.001 (Mann Whitney test). Raw data reported in S6 Table. Statistical tests performed on data grouped by biological replicate are reported in S11 Fig.

We performed electron microscopy to visualise changes in oocyst wall morphology that may not be visible by light microscopy. General morphology was observed using scanning electron microscopy (SEM). Transmission electron microscopy (TEM) was used to visualise changes to the inner and outer layers of the wall and to measure the thickness of these layers. By SEM, wild type and *∆cowp8* cannot be distinguished (Fig 4A and 4B). When quantified, both wild type and *∆cowp8* oocysts have similar proportions of oocysts appearing ‘wrinkled’ or ‘smooth’ (Fig 4C). ‘Wrinkles’ are an artefact of the resiliency of the oocyst from sample processing for SEM. This analysis is consistent with confocal microscopy which failed to identify morphological changes (Fig 3K–N). Cross sections of wild type and *∆cowp8* oocysts across two biological replicates were prepared for TEM (Fig 4D and 4E), and the thickness of the oocyst wall was measured. Despite loss of the abundant *COWP8*, *Δ*cowp8** oocyst walls were around ~12nm thicker than wild type (WT median wall width = 50.8nm and *Δ*cowp8** = 62.9nm; Fig 4F data plotted by biological replicate in S11 Fig).

It was surprising to find that *Δ*cowp8** oocysts maintain their thick wall. To investigate if parasites can compensate for loss of *COWP8*, for example by upregulating other members of the *COWP* family, oocysts were analysed by quantitative mass spectrometry. Wild type and *∆cowp8* oocysts were excysted, proteins were extracted, samples were labelled with tandem mass tags (TMTs) and then analysed using quantitative mass spectrometry. The amount of the other *COWP*s is not significantly changed upon loss of *COWP8* (S12 Fig and S7 Table).

### *Cowp8* mutants retain mechanical strength

To determine if there are changes to oocyst wall strength upon loss of *COWP8*, we measured *∆cowp8* oocyst walls using a biomechanical approach called nanoindentation. This approach is commonly used to measure the hardness and elastic modulus of biological materials [27]. Oocysts were immobilised on a petri dish and probed under a microscope. The probe approaches the sample at a constant speed, generating a force-displacement curve as the probe indents the sample with a defined indentation depth (5 µm). Young’s Modulus, which is the mechanical property measuring stiffness of a material by defining the relationship between stress and strain, can be obtained by fitting force-displacement curves to a corresponding mathematical model.

**Fig 5:**
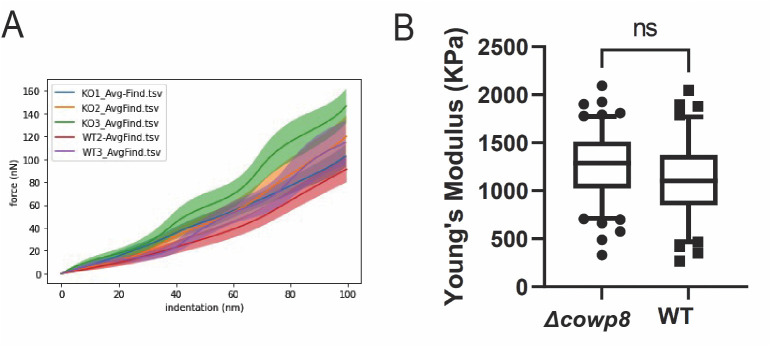
Oocysts lacking COWP8 maintain mechanical strength. A) Force indentation plot in range of 0-100 nm of indentation. Two technical replicates performed for wild type (purple and lilac; data from third wild type replicate was determined to be an outlier, see S13 Fig) and *∆cowp8* (blue, orange, and green). B) Young’s modulus was obtained from fitting the indentation plot to the Hertz mathematical model, producing box and whisker plots (10-90 percentile). Statistical analysis carried out on obtained data (Welch’s t-test) concluded that there is no significant difference in mechanical strength between wild type and *∆cowp8* oocysts (ns meaning p > 0.05), with an average Young’s Modulus of 1.2 MegaPascals (MPa) being calculated for both wild type and *∆cowp8* oocysts.

Oocysts were immobilised on a dish and subjected to nanoindentation using a Chiaro nanoindentor. From raw force displacement curves (S13A Fig), force indentation plots were created using average force measurements obtained from the first 100 nm of indentation (S13B Fig). Young’s Modulus was calculated by fitting the force indentation plots (Fig 5A) to the Hertz mathematical model for both wild type and *∆cowp8* oocysts. No difference in stiffness was observed and wild type and *∆cowp8* oocysts were determined to have an approximate average Young’s Modulus of 1.2 MegaPascals (MPa; Fig 5B). This is in range with previous measurements of the strength of *Toxoplasma gondii* oocyst walls at 10^6^ - 10^7^ MegaPascals (both sporulated and unsporulated oocysts measured) [28].

## Discussion

**Fig 6:**
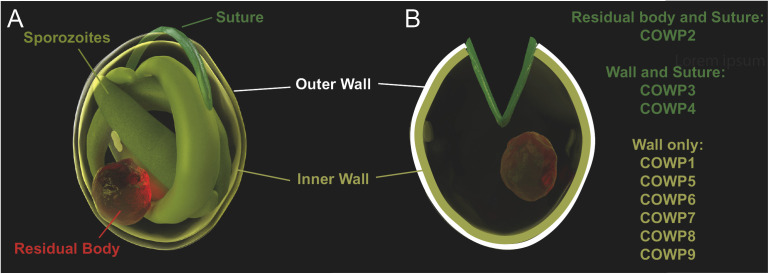
Model of *COWP*s in oocyst wall structure. A) 3D rendering of the *Cryptosporidium* oocyst structure, where 4 sporozoites (olive green) and a residual body (red) are tightly packed inside the slightly oval shell of the oocyst wall. Inner and outer walls are sealed closed by the suture (dark green). B) Upon excystation the suture opens, allowing sporozoites to escape often leaving behind the residual body inside the excysted oocyst wall. *COWP*s 1, 5, 6, 7, 8, 9 localise to the oocyst wall only. *COWP3* and *COWP4* localise to both the oocyst wall and the suture, while *COWP2* is localised to the residual body and the suture. Illustrations by Konstantinos Alexandrou.

Previously, only a single member of the *Cryptosporidium* Oocyst Wall Protein family was experimentally confirmed to be a true oocyst wall protein [19]. Here, using CRISPR, the remaining eight members of the *cowp* family were confirmed to be true oocyst wall proteins (Fig 6). Further, *COWP*s 2, 3, and 4 localised to a structure consistent with the size and shape of the oocyst suture. The suture forms a seam along the shorter end of the slightly oval-shaped oocyst. During the process of excystation, the suture opens allowing sporozoites to exit and infect the gut. *COWP* 2, 3, 4 are the first markers of this important structure. Why only some *COWP*s are destined to form the suture, and the others are localised across the oocyst wall is unclear. Additionally, *cowp2* was the only *cowp* that also localised to the residual body. This suggests that *cowp2* may play an important role in the construction of the suture, such that it is expressed beyond what is required, and excess protein aggregates within the residual body. Further investigation of these suture proteins will improve our understanding of the process by which the suture is constructed and how it is “opened” during excystation.

Although C-terminal tagging was attempted several times for *COWP*s 3, 4, and 5 (multiple independent transfections reported in S14 Fig), these strains were not recovered (when using both larger fluorescent tags, or smaller epitope tags on their own). It is likely because insertion of the tagging construct at the endogenous locus disrupted expression of the neighbouring gene and was not tolerated. Expression of these tagged *COWP*s using their corresponding or similar promoter, at a secondary location in the genome was well tolerated. This strategy can be employed in other instances where intergenic spaces are minimal and don’t allow modification of the original locus.

For the more highly expressed family members, *COWP6* and *COWP8*, their exclusive expression in macrogamonts was confirmed. Like *COWP1*, *COWP6* and *COWP8* are localised in multiple puncta throughout the cytoplasm of the macrogamont and resemble wall forming body organelles. New selection markers present an opportunity to create a reporter strain where more than one *COWP* is tagged, allowing colocalisation studies [29,30]. Immunoelectron microscopy could then be performed to localise *COWP*s to the wall forming bodies. Wall forming body (WFB) organelles have been observed using electron microscopy in female coccidia. Female parasites produce many of these membrane-bound organelles. Type I WFBs are electron dense and typically more spherical in shape. Type II WFBs are less electron dense, are more diverse in size, and more irregular in shape. In *Eimeria*, reporter proteins have been identified for each type of WFB [31]. In *Eimeria* it observed that after fertilisation, Type I WFB fuse with the parasite membrane and secrete their contents to the space between the parasite membrane and the host cell membrane. This material forms the outer layer of the oocyst wall. Type II WFB follow and secrete their contents next, forming the inner layer of the oocyst wall. We lack markers for Type I and Type II WFB in *Cryptosporidium*. However, as the outer layer of the *Cryptosporidium* oocyst wall is predominantly acid-fast lipids, it is possible these are packaged in Type I WFB and the *COWP*s and other associated proteins in Type II WFBs. More work is required to identify which category of WFB contain *COWP*s and to improve our understanding of the contribution of these organelles to construction of the oocyst wall.

*Cowp8* is a highly expressed member of the *cowp* family and is expressed last. This high and late level of expression suggest a unique role in oocyst construction and parasite transmission. Surprisingly, *∆cowp8* parasites are viable, infectious, and transmissible in standard laboratory settings. Similarly, we failed to observe changes in the thickness of the oocyst wall, its ability to be resistant to bleach, or its mechanical strength. Proteomics did not reveal changes in expression of any of the other members of the *cowp*s to compensate for a lack of *COWP8*. Therefore, while *COWP8* is a component of the oocyst wall, it is not essential for parasite survival, construction of the oocyst wall, or transmission. Given its high level of expression and uniquely “late” expression timing, the lack of essentiality of *cowp8* for survival or transmission was surprising. It is possible that *cowp8* is required for survival in more harsh environmental conditions that we cannot replicate in the laboratory (where mice are directly infected by gavage rather than from exposure in the environment). Or it is possible the function of *COWP8* is redundant with other members of the family, and *COWP1* or *COWP6* are sufficient. Improved understanding of how the *COWP*s interact and insight into if the other *cowp* family members are essential will provide further insight as to the function of *COWP8*.

In addition to the *cowp*s, other proteins are known to localise to the inner layer of the oocyst wall with acid-fast lipids forming the outer layer of the oocyst wall. The identity of these lipids and the process by which the two layers of the oocyst are formed is unknown. Although *COWP8* was not found to be essential for infection or transmission, we have developed a range of experimental techniques and assays to describe the phenotypes of *Cryptosporidium* mutant oocysts, including biomechanics. To our knowledge, this is the first time this has been applied to the study of *Cryptosporidium* oocysts and reported. These techniques provide a robust framework for further investigation of other components of the *Cryptosporidium* oocyst wall and for evaluating their role in construction of the oocyst wall and parasite transmission.

## Materials and methods

### Animal ethics

Mice randomly assigned to cages at the time of weaning. Both sexes were used for experiments described here. As required animals were sex and aged matched. All animal studies were ethically reviewed and carried out in accordance with Animals (Scientific Procedures) Act 1986 and the Dundee University Policy on the Care, Welfare, and Treatment of Animals. Regulated procedures on living animals were approved by the University’s Ethical Review Committee and carried out under the authority of project and personal licenses issued by the Home Office under the Animals (Scientific Procedures) Act 1986, as amended in 2012 (and in compliance with EU Directive EU/2010/63). The ERC has a general remit to develop and oversee policy on all aspects of the use of animals on University premises and is a subcommittee of the University Court, its highest governing body.

### Materials

Wild type *Cryptosporidium parvum* oocysts (Iowa strain) were purchased from Bunchgrass Farms (Deary, Idaho, USA) and stored at 4°C in PBS with penicillin/streptomycin. Oligonucleotides produced by either the University of Dundee Oligonucleotide Synthesis Service or Sigma-Aldrich (S1 Table). mScarlet-I described throughout is codon optimised for *C. parvum* (GeneScript; sequences available in S2 Table).

### Molecular cloning and DNA sequencing

PCR performed using high-fidelity DNA Polymerase (Primestar Max, Takara) and cloning with NEBuilder HiFi DNA Assembly (NEB). DNA Sequences confirmed via sequencing by Genewiz, University of Dundee MRC PPU Reagents and Services, or Plasmidsaurus. All plasmids created for this work listed in S2 Table with links available for DNA sequences.

Construct used for C-terminal tagging with mNeonGreen-3xHA previously described, referred to as mNeon throughout [29]. A second construct was created with mScarlet-I fused to a 3 × c-myc epitope tag followed by an NLuc-Neo^R^ fusion under the control of constitutively expressed *CpEnolase* promoter to generate “pLIC-mScarlet-I-3xc-myc_aldo 3´utr_ENNE”. Construct for deletion of *Cpcowp8* contains mScarlet-I, 2A linker, and an NLuc-Neo^R^ fusion, all of which is under the control of the *CpEnolase* promoter to generate “EnoP_mScarlet-I-ENNE”.

Due to the location of the gRNA at the C-terminus, the last 129 bp of *COWP4* was cloned into a tagging construct fused directly to the mNeon tagging construct. The codon for cysteine at amino acid position 845 was mutated from “TGC” to “TGT” to prevent further cutting by Cas9 to generate “*COWP4* C-term-mNeon-3×HA_ENNE”. Similarly, the last 114 bp of *COWP9* was cloned into a tagging construct fused directly to an mNeon-3 × HA. The codon for lysine at amino acid position 1180 was mutated from “AAG” to “AAA” to prevent further cutting by Cas9 to generate “*COWP9* C-term-mNeon-3×HA_ENNE”.

To express *COWP3*, *COWP4*, *COWP5* under their endogenous promoter from an ectopic location, the entire intergenic region upstream of the corresponding *cowp* gene and its full open reading frame (except the stop codon) was amplified using PCR from *C. parvum* genomic DNA. This was cloned to generate “*Cowp*3P_COWP3ORF-mNeon-3×HA_ENNE,” “*Cowp*4P_COWP4ORF-mNeon-3×HA_ENNE,” and “*Cowp*5P_COWP5ORF-mNeon-3xHA_ENNE”.

Guide oligonucleotides were designed for *COWPs* 2–9 (S1 Table) and ligated into a plasmid containing Cas9/U6 promoter containing plasmid [32] using restriction enzyme cloning as previously described [33]. To generate the *Δ*impdh::COWP3-mNeon**, *Δ*impdh::COW4-mNeon**, and *Δ*impdh::COWP5-mNeon**, a guide for the inosine monophosphate dehydrogenase (*IMPDH*, cgd6_20) locus was used [34].

### CRISPR design

Linear repair cassettes were generated via PCR using PrimeSTAR MAX DNA polymerase (Takara). Fifty bp of homology, typically directly flanking the site targeted by CRISPR, was incorporated on primer.

To generate COWP2-mNeon, *cowp2* (cgd7_1800) was targeted by a gRNA located 38 bp upstream of the stop codon. Regions of homology consisting of the 68 bp directly upstream of the stop codon, and 50 bp located 116 bp downstream of the stop codon. A synonymous mutation of serine at position 1373 encoding the PAM (AGG to AGA) was engineered to prevent further targeting of Cas9 upon integration of the repair cassette into the genome.

To attempt to generate COWP3-mNeon and COWP3-HA, *cowp3* (cgd4_670) was targeted by a gRNA located 16 bp after the stop codon. Regions of homology consisting of the 50 bp directly upstream of the stop codon, and 50 bp located 177 bp downstream of the stop codon.

To attempt to generate COWP4-mNeon, *cowp4* (cgd8_3350) was targeted by a gRNA located 124 bp after the stop codon. Regions of homology consisting of the 179 bp upstream of the stop codon, and 50 bp located 153 bp downstream of the stop codon.

To attempt to generate COWP5-mNeon and COWP5-HA, *cowp5* (cgd7_5150) was targeted by a gRNA located 17 bp upstream of the stop codon. Regions of homology consisting of the 71 bp upstream of the stop codon, and 50 bp located 130 bp downstream of the stop codon. A synonymous mutation of threonine at position 623 encoding the PAM (CGG to CTG) was engineered to prevent further targeting of Cas9 upon integration of the repair cassette into the genome.

To generate *Δ*impdh::COWP3-mNeon**, *Δ*impdh::COWP4-mNeon**, *Δ*impdh::COWP5-mNeon**, and *Δ*impdh::cowp1P-COWP5-mNeon** the *CpIMPDH* locus was targeted using CRISPR as previously described [34].

To generate COWP6-mScarlet-I, *cowp6* (cgd4_3090) was targeted by a gRNA located 13 bp downstream of the stop codon. Regions of homology consisting of the 57 bp directly upstream of the stop codon, and 50 bp located 54 bp downstream of the stop codon. A synonymous mutation of serine at amino acid position 536 encoding the PAM (AGC to AGT) was engineered to prevent further targeting of Cas9 upon integration of the repair cassette into the genome.

To generate COWP7-mNeon, *cowp7* (cgd4_500) was targeted with a guide RNA located 19 bp upstream of the stop codon. Homology regions of 92 bp located directly upstream from the stop codon, and 50 bp located 56 bp downstream of the stop codon. The PAM was mutated such that proline at position 827 was replaced with alanine.

To generate COWP8-mNeon, *cowp8* (cgd6_200) was targeted with a guide RNA found 39 bp upstream of the stop codon. Homology regions of 105 bp located directly upstream of the stop codon, and 50 bp located 286 bp downstream of the stop codon within the 3´ UTR. The PAM was mutated such that glycine at position 452 was replaced with alanine. The same guide RNA and downstream homology was utilised in the knockout strategy. New upstream homology was selected, consisting of 50 bp located 271 bp upstream of the *cowp8* gene start codon.

The same gRNA and downstream homology for *CpCOWP8* C-terminal tagging was used to generate *Δ*cowp8**. New upstream homology was selected, consisting of 50 bp located 271 bp upstream of the *CpCOWP8* start codon.

To generate COWP9-mScarlet-I, *cowp9* (cgd6_210) was targeted with a guide RNA located 31 bp upstream of the stop codon. Upstream homology was already cloned into the tagging construct. Downstream homology of 50 bp located 266 bp after the stop codon.

### Excystation

Wild type *C. parvum* oocysts were incubated in 4% bleach on ice for 5 minutes and then washed three times with PBS. Treatment with bleach serves to kill residual bacteria and remove faecal contaminants; this also disrupts the “outer veil” which may be observed otherwise without bleach treatment [11]. Oocysts were incubated for 1 hour at 37°C in either 0.2 mM sodium deoxytaurocholate in PBS [33], 0.5% (w/v) taurodeoxycholic acid in 0.25% trypsin in PBS [35], or 0.75% sodium taurocholate (Merck) in RPMI-1640 medium with 1% foetal bovine serum [36] to induce excystation.

### Propagation of transgenic strains in immunocompromised mice

IFN-gamma KO mice (Jackson Laboratory #B6.129S7-IfngtmlTS/J, JAX 002287 bred at the University of Dundee; at least 8 weeks old) were given antibiotics in the drinking water (1 mg/ml ampicillin, 1 mg/ml streptomycin, 0.5 mg/ml vancomycin final concentrations) for 1 week prior to infection. Excysted sporozoites were transfected using a 4D Amaxa Nucleofector as previously described [33]. *cowp1P_COWP5-mNeon* strain transfected using a 4D Amaxa with modifications as described [36]. Mice were gavaged with saturated sodium bicarbonate 5 min prior to infection by gavage with transfected sporozoites. This serves to neutralise stomach acid before gavage with sporozoites and enhance parasite survival and transit to the intestines. Both sexes used to generate new strains of transgenic *Cryptosporidium* (2 or 4 mice). We have found that two mice are sufficient to generate new strains of *Cryptosporidium*. This reduction in animals was specifically utilised to produce transgenic COWP2-HA and *Δ*impdh::COWP3-mNeon**, *Δ*impdh::COW4-mNeon**, and *Δ*impdh::COWP5-mNeon**. Paromomycin (16 g/L) was added to the drinking water for the duration of the infection, or as indicated.

To passage transgenic strains, mice were gavaged with faecal slurry or purified oocysts. Mice were treated with selection agents as indicated. Faecal samples were collected post infection as indicated and were pooled from the cage of mice.

### Faecal sample analysis

NanoLuciferase assay was performed on 20 mg of homogenised faecal sample (in 1mL of lysis buffer). 25 µL of NanoLuciferase substrate-lysis buffer mixture was added to 100 µL of faecal lysate (GloMax plate reader, Promega). Positive threshold for NanoLuciferase signal is RLU/mg > 500. Oocysts were purified from faecal samples using sucrose and Caesium chloride flotations as previously described [33].

DNA was extracted from either ~100 mg faecal material or purified oocysts. Samples were subjected to minimum of five freeze thaw cycles and then DNA was extracted using either a Zymo Quick-DNA Faecal/Soil Microbe DNA Miniprep Kit (faecal sample) or a Qiagen DNeasy Blood & Tissue Kit (oocysts). DNA was amplified by PCR using primers specific to the 5´ and 3´ regions of the repair cassette integration site. For knockout mutants, a third set of primers designed to amplify within the targeted genes ORF were also used. *α-tubulin* served as the positive control.

### Whole genome sequencing and analysis

In the case of *Δ*cowp8**, DNA was extracted from 10 million purified oocysts and sequenced and analysed by University of Glasgow Polyomics. Briefly, library was prepared using NEBNext UItra II FS DNA kit, which uses enzymatic fragmentation. Library was then sequenced pair-ended reads of 100 bp to an average of >5 million reads on an Illumina NextSeq2000. The FASTQ files forward and reverse paired-end reads of *Δ*cowp8** sample were aligned to the reference genome v68 of *C. parvum* clone IOWAII downloaded from CryptoDB [37] using Bowtie2 version 2.3.5 [38], with the ‘very-sensitive-local’ pre-set alignment option. The alignments were converted to BAM format, reference sorted and indexed with SAMtools version 1.9 [39]. The genome coverage of the aligned reads was extracted from the BAM files using bedtools version v2.29.0 [40] with the -pc option for pair end data and -bg option to output a bedGraph file. The GFF annotation file for *C. parvum* clone IOWAII was downloaded from CryptoDB and converted to GTF with gffread (https://github.com/gpertea/gffread). The bedGraph file and GTF file were visualized with the svist4get python package version 1.2.24 [41].

### Fluorescence microscopy

#### Live oocysts.

Live oocysts were bleached, washed, and resuspended in a 1:1 mix of matrigel (Corning):PBS. Oocysts were loaded onto a µ-Slide Angiogenesis slide (Ibidi, catalog #81501) and allowed to settle overnight at 4˚C. Oocyst:matrigel mixture was incubated at 37˚C for 15 minutes immediately before imaging to solidify matrigel and immobilize oocysts.

#### Excysted oocysts.

Two million oocysts were incubated in 4% bleach on ice for 5 minutes and then washed three times with PBS. Oocysts were incubated overnight at 37°C (15 hours) in 0.75% Sodium taurocholate in RPMI-1640 medium (Gibco) with 1% foetal bovine serum. If necessary, parasites underwent 10 freeze-thaw cycles (liquid nitrogen to 37°C) to aid excystation. Oocysts were then washed once in PBS and fixed for 10 min in 4% paraformaldehyde in PBS. After a PBS wash, samples were permeabilised with 0.25% Triton X-100 in PBS for 10 min. Samples were then blocked using 3% bovine serum albumin (BSA) (w/v) and 12% normal goat serum (Sigma) in PBS for one hour shaking at 300rpm at room temperature. Oocysts were labelled with primary antibody (antibodies prepared in 1% BSA and 0.05% Triton-X-100 in PBS) for 1 hour. Samples were washed with 1% BSA and 0.05% Triton-X-100 in PBS and stained with secondary antibodies for 1 hour. After a final wash, oocysts were resuspended in 20 μL of matrigel (Corning) and loaded onto a µ-Slide Angiogenesis slide (Ibidi, catalog #81501) and allowed to settle overnight at 4˚C.

#### Intracellular stages.

HCT-8 cells (ATCC catalogue #CCL-244; RRID: CVCL_2478) were cultured on sterile 12mm glass coverslips in a 24-well plate. Once cells reached a confluency between 40–60%, bleached and washed oocysts were added. Cultures were fixed at the indicated time points with 3% paraformaldehyde, quenched with 125 mM glycine and permeabilized with 0.05% saponin in PBS (10 minutes). Samples were labelled as described for excysted oocysts.

Intestinal tissue was harvested at peak infection. The distal ileum was flushed with PBS and fixed overnight, gently rocking in 4% (w/v) paraformaldehyde at 4˚C. Tissue was dehydrated in 30% (w/v) sucrose in PBS overnight at 4˚C and embedded in OCT compound (Agar Scientific) and cryosectioned (10 µm thick, Leica CM1850 Cryostat). Sections were dried overnight onto Thermo Scientific SuperFrost Plus Adhesion slides. Samples were stained with Hoechst 33342 (Thermo Scientific) and Phalloidin-647 (Abcam, catalog #ab176759) and mounted using ProLong Glass Antifade (Invitrogen).

#### Fluorescence imaging.

All antibodies and dyes used are described in S3 Table. All images collected at the University of Dundee Imaging Facility; the Open Microscope Environment (OMERO) was utilised for image management. Z-stacked images of tissue sections were acquired using a DeltaVision Widefield Deconvolution microscope or a Zeiss LSM 880 (optionally with Airyscan detector) as indicated.

### SEM and TEM microscopy

For routine transmission electron microscopy and immunostained electron microscopy (immunoEM), four million oocysts (wild type or COWP8-mNeon) were bleached and washed three times in PBS (excluding *Δ*cowp8** oocysts). All oocyst samples were fixed overnight at 4 °C in EM-grade 4% paraformaldehyde and either 2.5% (for standard TEM) or 0.2% (for immunogold TEM) glutaraldehyde in PBS. Fixed parasites were sent for processing and imaging at the Cellular Analysis Facility, University of Glasgow.

For routine TEM, oocysts were washed in 0.1 M phosphate buffer, before being post-fixed in 1% OsO_4_ and 1.25% Potassium ferrocyanide (vol:vol) for 1 hour on ice in darkness. Oocysts were then washed in distilled water following contrast en bloc with 0.5% aqueous uranyl acetate for 1 hour at room temperature in darkness. Samples were then dehydrated in an ascending series of acetone and embedded in epoxy resin. Ultra-thin sections (60 nm) were cut and collected onto formvar-coated copper grids and contrasted with 4% Uranyl acetate prior imaging using a JEOL 1200 EX transmission electron microscope, operating at 80kV. *Δ*cowp8** oocysts were pooled from passages two, three, five, eight, nine and ten for the first TEM sample preparation. For the second repeat, *Δ*cowp8** oocysts were purified from passage 10 in NOD-SCID mice. Wild type oocysts were used from the same isolation from Bunchgrass Farms in January 2024. Two independent sample preparations were analysed and compared.

For immunoEM, after several washes in the cacodylate 0.1M buffer, the samples were dehydrated in ascending ethanol series and embedded in LR White resin (Agar Scientific). Ultrathin sections (60 nm thick) were obtained using an ultramicrotome (Leica Microsystems). The sections were collected on formvar-coated nickel grids and then blocked in PBS containing 3% bovine serum albumin for 1 hour. After this time, they were incubated in the presence of primary antibody (either a monoclonal mouse anti-c-myc (ProteinTech) or a monoclonal rat anti-HA (SigmaAldrich)). Then they were washed several times in blocking buffer and incubated with 15 nm gold-conjugated Protein A (Aurion). The grids were washed several times in the blocking buffer, dried and contrasted with 4% Uranyl acetate, and observed using a JEOL 1200 EX transmission electron microscope operating at 80kV.

For scanning electron microscopy two million oocysts were bleached and washed three times in PBS. Oocysts were fixed at 4 °C overnight in EM-grade 4% paraformaldehyde and 2.5% glutaraldehyde in PBS. Fixed oocysts were washed in 0.1 M phosphate buffer, then left on top of poly-l-lysine coated glass slides for 20 min to allow oocysts to adhere. Adhered oocysts were washed in 0.1 M phosphate buffer, before being dehydrated in an ascending series of ethanol concentrations and finally reaching critical point dehydration with a Autosamdri-815 machine (Tousimis). Dried coverslips were coated with gold/palladium (10 nm thick layer) and imaged in a JEOL IT-100 scanning electron microscope. A minimum of 10 fields of view for each sample were used for quantitation.

### TEM analysis

Levels of grey images were processed with a gaussian blur filter prior to thresholding by eye (Otsu method) to create a binary image of the oocyst wall. Where sporozoite material was attached to the oocyst wall this was manually broken using the pencil tool. The oocyst wall ROI was used to create a mask. A centreline was added prior to running the Voronoi width tool using the MorphoLibJ-plugin [42] and width_profile_tools toolset [2] (available on github; see software section of Methods). This generated the width-profile of the oocyst wall calculated as Voronoi distance from line-segments perpendicular to the centreline of the wall. Line segments were measured every pixel (equivalent to a range of between 58–344 nm dependent on the scale of the image analysed).

Images were excluded from analysis where the whole width of the oocyst wall was not captured completely at the edges, or where accurate thresholding of the image was prevented, either by artefacts or by the oocyst wall touching sporozoite material extensively. For some images, the oocyst wall was split into multiple masks to avoid artefacts. Values across all masks generated from one oocyst were combined for analysis. Where more than one oocyst was captured in an image, width measurements were analysed separately.

In biological replicate one 51 images of wild type oocysts were collected. Of these, 14 whole oocysts were analysed (three images excluded) and 34 zoomed in images were collected and analysed. 23 images of *Δ*cowp8** were collected. 5 whole oocysts and 16 zoomed in images were analysed (three images excluded). In biological replicate two, 10 images of WT and 10 images of *Δ*cowp8** were collected. Three *Δ*cowp8** images were excluded. 10 whole WT oocyst walls and 10 whole *Δ*cowp8** oocyst walls were measured.

### *In vivo* phenotyping experiments

25,000 oocysts were resuspended in PBS and exposed to 60°C in an Eppendorf ThermoMixer. A temperature of 60 °C was chosen, as this temperature is at the lower end of reported temperatures known to heat inactivate *Cryptosporidium* [43–45]. Samples were then stored at 4 °C overnight.

50,000 oocysts were resuspended in either 0% or 8% undiluted bleach solution and incubated on ice for 5 minutes. Oocysts were washed in PBS three times and then stored at 4 °C overnight.

Age and sex matched interferon-gamma knockout mice (4 mice were cage) were pre-treated prior to infection with 5,000 oocysts per strain by oral gavage.

### Nanoindentation

Petri dishes (35 mm × 10 mm, Corning cat no. 430588) were treated with Corning Cell-Tak cell and tissue adhesive (cat no. CLS354240) the day before oocyst attachment. Cell-Tak was added to 0.1 M Sodium bicarbonate solution, applied to 35 mm dish, and incubated for a minimum of 20 min at room temperature. Cell-Tak solution was aspirated and dish washed twice with sterile water. Dishes were left to air dry and stored in at 4 °C.

One million *Δ*cowp8** and WT oocysts in PBS were applied to a dish and incubated overnight at 4 °C. Dishes were gently washed once with sterile PBS and stored in sterile PBS to prevent oocysts desiccation. Nanoindentation measurements and analysis were conducted at the Advanced Research Centre (ARC), University of Glasgow. Nanoindentation measurements were performed using a Chiaro nanoindenter (Optics 11) mounted on top of an inverted phase contrast microscope (Evos XL Core, Thermofisher), following a previously described approach [46]. Measurements were performed at room temperature in PBS. Each sample was indented once with a spherical tip of 3 μm radius at a speed of 2 μm/s over a vertical range of 5 μm. Measurements were performed on oocysts adhered to three separates petri dishes, sampling a minimum of ten oocysts per plate (n = 60–67) and the experiment was repeated twice (N = 2).

The selected cantilever had a stiffness of 4.18 N/m. The collected curves were analysed using a custom Python code [47]. Curves were first aligned using a baseline detection method based on the histogram of the force signal [48] and the corresponding indentation was calculated for each curve. The analysis was performed using the Hertz model for a spherical indenter to fit the curves obtained.

## Proteomics

### Sample preparation and protein extraction

Samples of wild type oocysts and *Δ*cowp8** oocysts were prepared as two biological repeats (with two technical repeats each) and analyzed using quantitative proteomics. 40 million oocysts per technical repeat were bleached, washed three times in PBS, and excysted at 37 °C in 0.2 mM Sodium taurochlorate for 2 hours. Excysted material was resuspended in 25 µL lysis buffer (4% SDS, 10 mM DTT, 1x protease inhibitor cocktail, all prepared in water). Samples were subjected to five cycles of freeze thaw to excyst remaining oocysts. Samples were incubated at 56 °C for 1 hour while shaking (800 rpm) to solubilise proteins. Samples were centrifuged at 20,000xg for 40 minutes at 4 °C. Supernatant was removed and TCEP *(*tris(2-carboxyethyl)phosphine*)* added to a final concentration of 25 mM and incubated at 37 ºC for 10 minutes. Added IAA (iodoacetamide) to a final concentration of 25 mM and incubated at room temperature in the dark for 1 hour. Added 13% volume ice-cold TCA and store at -20 C. Sample was centrifuged at 16,000 xg for 4 °C for 5 minutes. Supernatant was removed and pellet was washed with 0.5 ml cold acetone. Acetone wash repeated a total of five times and then the sample was air dried. Protein extract was resuspended in 400 µl TEAB, sonicated to resuspend the material. Sample was digested with Trypsin/LysC (Promega) overnight at 37 ºC with shaking and then dried.

### TMT labelling and high pH reverse phase fractionation

Tryptic peptides (11.6 µg, from each sample) were dissolved in 100 µl of 150 mM TEAB. TMT labelling was performed according to the manufacturer’s instructions (Thermo-Fisher Scientific). The different TMT-10 plex labels (0.8 mg) (Thermo Fisher Scientific) were dissolved in 41 µl of anhydrous acetonitrile, and each label is added to a different sample. The mixture was incubated for 1 hour at room temperature, an equivalent of 0.75 µg of peptides from each sample were mixed with 20 µl 1% formic acid and used to check labelling efficiency. The remaining samples were kept at –80 °C, until the labelling efficiency was checked. Samples were pooled, desalted, and dried in a speed-vac at 30 °C. Samples were re-dissolved in 200 µl Ammonium formate (10 mM, pH 9.5) and peptides were fractionated using High pH RP Chromatography. A C18 Column from Waters (XBridge peptide BEH, 130 Å, 3.5 µm 2.1 X 150 mm, Waters, Ireland) with a guard column (XBridge, C18, 3.5 µm, 2.1 X 10mm, Waters) were used on an Ultimate 3000 HPLC (Thermo-Scientific). Buffers A and B used for fractionation consist, respectively, of (A) 10 mM ammonium formate in milliQ water pH 9.5 and (B) 10 mM Ammonium formate, pH 9.5 in 90% acetonitrile. Fractions were collected using a WPS-3000FC auto-sampler (Thermo-Scientific) at 1minute intervals. Column and guard column were equilibrated with 2% Buffer B for twenty minutes at a constant flow rate of 0.2 ml/min. Fractionation of TMT labelled peptides was performed as follows; 190 µl aliquot were injected onto the column, and the separation gradient was started 1 minute after the sample was loaded onto the column. Peptides were eluted from the column with a gradient of 2% Buffer B to 20% Buffer B in 6 minutes, then from 20% Buffer B to 45% Buffer B in 51 minutes and finally from 45% buffer B to 100% Buffer B within 1 min. The column was washed for 15 minutes in 100% Buffer B. The fraction collection started 1 minute after injection and stopped after 80 minutes (total 80 fractions, 200 µl each). Formic acid (30 µl of 10% stock) was added to each fraction and concatenated in groups of 20 fractions.

### LC-MS analysis

Analysis of peptides was performed on a Q-exactive-HF (Thermo Scientific) mass spectrometer coupled with a Dionex Ultimate 3000 RS (Thermo Scientific). LC buffers were the following: buffer A (0.1% formic acid in Milli-Q water (v/v), buffer B (80% acetonitrile and 0.1% formic acid in Milli-Q water (v/v) and loading buffer (0.1% TFA).

Peptides from each fraction were resuspended in 50 µl 1% formic acid and aliquots of 5 μl were loaded at 10 μL/min onto a trap column (100 μm × 2 cm, PepMap nanoViper C18 column, 5 μm, 100 Å, Thermo Scientific) equilibrated in 0.1% TFA. The trap column was washed for 5 min at the same flow rate with 0.1% TFA and then switched in-line with a Thermo Scientific, resolving C18 column (75 μm × 50 cm, PepMap RSLC C18 column, 2 μm, 100 Å) equilibrated in 5% buffer B for 17 min. The peptides were eluted from the column at a constant flow rate of 300 nl/min with a linear gradient from 5% buffer B (for Fractions 1–10, 7% for Fractions 11–20) to 35% buffer B in 125 min, and then from 35% buffer B to 98% buffer B in 2 min. The column was then washed with 98% buffer B for 20 min and re-equilibrated in 5% or 7% buffer B for 17 min. The column was maintained at a constant temperature of 50 ^°^C.

Q-exactive HF was operated in data dependent positive ionisation mode. The source voltage was set to 2.85 Kv and the capillary temperature was 250 ^°^C. A scan cycle comprised MS1 scan (m/z range from 335-1600, with a maximum ion injection time of 50 ms, a resolution of 120 000 and automatic gain control (AGC) value of 3x10^6^) followed by 15 sequential dependant MS2 scans (resolution 60000) of the most intense ions fulfilling predefined selection criteria (AGC 1x10^5^, maximum ion injection time 200 ms, isolation window of 0.7 m/z, fixed first mass of 100 m/z, spectrum data type: centroid, intensity threshold 5 x 10^4^, exclusion of unassigned, singly and >6 charged precursors, peptide match preferred, exclude isotopes on, dynamic exclusion time of 45 s). The HCD collision energy was set to 32% of the normalised collision energy. Mass accuracy is checked before the start of samples analysis.

### Quantitative proteomics analysis of transgenic *Cryptosporidium*

Proteomic data were processed through MaxQuant software (version 2.4.10.0), leveraging its integrated Andromeda search engine [49]. The search database was specifically constructed for *Cryptosporidium parvum* Iowa II, with annotated protein sequences obtained from CryptoDB [37], release 61. This was augmented with sequences for the reporter proteins mScarlet-I and NLuc-Neo^R^. Additionally, a murine protein database, retrieved from Uniprot [50] on January 20, 2023, was concatenated with the *C. parvum* database to account for potential host protein contamination. The analysis encompassed both TMT 6-plex and TMT 10-plex labeling, processed in parallel within a single MaxQuant instance. Each TMT experiment was treated as a distinct analytical batch, with no normalization applied between them. Trypsin was designated as the proteolytic enzyme, with a specification for up to two missed cleavages per peptide allowed. Carbamidomethylation of cysteine residues was set as a constant post-translational modification (PTM), whereas N-terminal acetylation of proteins and methionine oxidation were configured as variable PTMs. Default settings were retained for all other parameters within MaxQuant, except for TMT label correction factors. These were adjusted according to the manufacturer’s instructions (Thermo Fisher Scientific) and are detailed within the MaxQuant parameter files. These files, alongside the raw data, have been submitted to the PRIDE database. Data is available under Project accession: PXD050089.

Pre-normalization, we filtered the MaxQuant output to exclude potential confounders. Data were refined by removing entries solely identified by peptide modification sites (only identified by site), entries marked as reverse database matches (reverse), and proteins classified as potential contaminants (potential contaminant). For our analysis of *Cryptosporidium parvum* COWP8 across wild type (WT) and knockout (KO) specimens, we used a robust normalization technique tailored specifically for TMT proteomic data. Our experimental set consisted of one biological replicate with two technical replicates integrated within a TMT 6-plex configuration, along with an additional biological replicate paired with two technical replicates in a TMT 10-plex setup. To normalize the signal of the two TMT batches, we adopted a modified version of the Internal Reference Standard (IRS) normalization method, as initially delineated by Plubell et al. [51] The IRS normalization was applied by taking the raw mean intensity of each plex to serve as the reference channel. We computed the sum of the intensities for each TMT channel row-wise and then calculated the geometric mean of these sums to establish a stable reference point. This method was chosen to replace the reference channel utilized in [51], which was not generated from our datasets. The scaling factors for normalization were ascertained by dividing this geometric mean by the sum intensities of each individual experiment. Subsequently, each plex underwent individual scaling to ensure uniformity in signal intensities. Upon normalizing the data, we employed Principal Component Analysis (PCA) to verify the efficacy of the IRS process between the TMT 6-plex and 10-plex setups. The differential expression analysis was performed with the limma package [52] using the WT samples versus the KO samples, with log2 values. FDR values were computed with the toptable function in limma. The output table of the analysis is available as attached csv file (S7 Table).

### Statistics and software

Graphs and all statistical analyses were prepared using GraphPad Prism version 10.2.1. Mann-Whitney U Test was used to measure differences between median oocyst wall width values between two parasite strains (Fig 4F). Kruskal Wallis multiple comparisons test applied to compare median oocyst wall width values between two biological replicates of the two parasite strains (S11 Fig). Standard t-test was used to measure differences in Young’s modulus between two parasite strains, and Welch’s correction was applied due to differences in sample size (Fig 5B). Oocyst wall width measured using “Width Profile Tools” available at: https://github.com/MontpellierRessourcesImagerie/imagej_macros_and_scripts/wiki/Width-Profile-Tools. Curves generated for biomechanics measurements were analysed using a custom Python code as described above. Proteomic data were processed through MaxQuant software (version 2.4.10.0). The differential expression analysis was performed with the limma package as described above. These files, alongside the raw data, have been submitted to the PRIDE database; data is available under Project accession: PXD050089.

## Supporting information

S1 Fig Generation of *COWP2* tagged strain. A) gRNA (black arrow) and regions of 50 bp of homology (grey) were used to target the C-terminus of *COWP2* (cgd7_1800) for fusion with mNeon (mNeon in yellow and 3 × HA in orange) or the simplified 3 × HA tag alone. Both strains include NanoLuciferase-Neomycin resistance fusion protein (NLuc-Neo^R^) expressed by the constitutive *CpEnolase* promoter. B) Infection level of mice as measured by faecal NLuc, limit of detection at 500 RLU/mg, dotted line. Average and SD of three technical replicates of one biological replicate. The first passage of COWP2-mNeon (black circles) was well above the limit of detection. The first passage of COWP2-HA (1’ white circles) and the second passage in mice (2’ white squares) both established robust infections. C-D) PCR with primer pairs indicated in (A) was performed using genomic DNA extracted from wild type and transgenic strains. PCR products confirm correct integration of (C) mNeonGreen or (D) HA repair cassette at the C-terminus of *Cp*COWP2.
(TIF)

S2 Fig Expression of COWP3-mNeon, COWP4-mNeon and COWP5-mNeon tagged strains at *CpIMPDH* locus. A) Promoter and full open reading frame (ORF) of *COWP3*, *COWP4* and *COWP5* each cloned with C-terminal mNeonGreen to generate COWP3/4/5-mNeon repair cassette. Each repair cassette was targeted for integration at *Cpimpdh* locus (cgd6_20) using gRNA (black arrow) and regions of 50 bp of homology (grey). B) Infection level of mice as measured by faecal NLuc, limit of detection at 500 RLU/mg, dotted line. Average and SD of three technical replicates of one biological replicate. The first passage of *∆impdh*::COWP3-mNeon (black circles), *∆impdh*::COWP4-mNeon (white circles) and *∆impdh*::COWP5-mNeon (white squares) were all well above the limit of detection. C-E) PCR with primer pairs indicated in (A) was performed using genomic DNA extracted from wild type and *∆impdh*::COWP3-mNeon (C) *∆impdh*::COWP4-mNeon and (D) *∆impdh*::COWP5-mNeon (E).
(TIF)

S3 Fig Generation of COWP6-mScarlet-I and COWP7-mScarlet-I strains. A) Strategy to target the C-terminus of *COWP6* (cgd4_3090) for fusion with the mScarlet-I-3 × myc Repair Cassette (mScarlet-I in magenta and 3 × myc in blue). NanoLuciferase-Neomycin resistance fusion protein (NLuc-Neo^R^) expressed by the constitutive Cp*Enolase* promoter. gRNA (black arrow) and regions of 50 bp of homology (grey). B) Infection level of mice as measured by faecal NLuc, limit of detection at 500 RLU/mg, dotted line. Average and SD of three technical replicates of one biological replicate. The first passage of COWP6-mScarlet-I (black circles) was well above the limit of detection. C) PCR with primer pairs indicated in (A) was performed using genomic DNA extracted from wild type and COWP6-mScarlet-I. D) Strategy to target the C-terminus of *COWP7* (cgd4_500) for fusion with the mScarlet-I-3 × myc Repair Cassette (same as reported in A). *Cowp7* is predicted to contain 8 introns; exons indicated by white boxes. NanoLuciferase-Neomycin resistance fusion protein (NLuc-Neo^R^) expressed by the constitutive Cp*Enolase* promoter. gRNA (black arrow) and regions of 50 bp of homology (grey). E) Infection level of mice as measured by faecal NLuc, limit of detection at 500 RLU/mg, dotted line. Average and SD of three technical replicates of one biological replicate. The first passage of COWP7-mScarlet-I (black circles) was well above the limit of detection. F) PCR with primer pairs indicated in (D) was performed using genomic DNA extracted from wild type and COWP7-mScarlet-I.
(TIF)

S4 Fig Generation of COWP8-mNeon and COWP9-mNeon strains. A) Strategy to target the C-terminus of *COWP8* (cgd6_200) for fusion with the mNeon-3xHA Repair Cassette (mNeon in yellow and 3 × HA in orange). NanoLuciferase-Neomycin resistance fusion protein (NLuc-Neo^R^) expressed by the constitutive Cp*Enolase* promoter. gRNA (black arrow) and regions of 50 bp of homology (grey). Note that neighbouring gene upstream of *cowp8* is *cowp9*, illustrated in black. Mouse infections reported in Fig 3A–F. B) PCR with primer pairs indicated in (A) was performed using genomic DNA extracted from wild type and COWP8-mNeon. C) Strategy to target the C-terminus of *COWP9* (cgd6_210) for fusion with the mNeon-3 × HA Repair Cassette (same as in A). gRNA (black arrow) and regions of 50 bp of homology (grey). Note that neighbouring gene downstream of *cowp9* is *cowp8*, illustrated in black. D) Infection level of mice as measured by faecal NLuc, limit of detection at 500 RLU/mg, dotted line. Average and SD of three technical replicates of one biological replicate. The first passage of COWP9-mScarlet-I (black circles) was well above the limit of detection. E) PCR with primer pairs indicated in (C) was performed using genomic DNA extracted from wild type and COWP9-mNeon.
(TIF)

S5 Fig Immunoelectron microscopy of COWP6-mScarlet and COWP8mNeon. A) Immunoelectron microscopy of COWP6-mScarlet-I confirm localization to the inner layer of the oocyst wall. Representative image shown. B) Immunoelectron microscopy of COWP8-mNeon confirm localization to the inner layer of the oocyst wall. Representative image shown. C) A second localisation in globular-type structures (globule does not appear to be membrane bound) in the space between the sporozoite membrane and inside of the oocyst wall was observed to be positive for *COWP8* localisation by Immunoelectron microscopy. Representative image shown. Quantitation provided in S4 Table.
(TIF)

S6 Fig Expression of *COWP*s as determined from bulk RNA Sequencing of macrogamont life cycle stages. Transcript level of members of the *cowp* family from “female *in vivo*” sample from [23] as published on CryptoDB.org [53].
(TIF)

S7 Fig Reporter strain of COWP5-mNeon tagged strains at *CpIMPDH* locus. A) The Promoter of *cowp1* [23] and full open reading frame (ORF) of *cowp5* was cloned with C-terminal mNeonGreen to generate COWP5-mNeon repair cassette. Each repair cassette was targeted for integration at *Cpimpdh* locus (cgd6_20) using gRNA (black arrow) and regions of 50 bp of homology (grey). B) Infection level of mice as measured by faecal NLuc, limit of detection at 500 RLU/mg, dotted line. Average and SD of three technical replicates of one biological replicate. C) PCR with primer pairs indicated in (A) was performed using genomic DNA extracted from wild type and *∆impdh*::*cowp1P_*COWP5-mNeon. D) *∆impdh*::COWP5-mNeon fluorescence microscopy of live oocysts confirms that COWP5 localises to the oocyst wall. Confocal, single z-plane, brightfield: exposure time 0.18 msec, laser power 0.85%. mNeon: exposure time 0.18 msec, laser power 0.85%.
(TIF)

S8 Fig COWP2-mNeon and COWP2-HA localise to the residual body and suture. A) COWP2-mNeon oocysts hatched and stained with primary and secondary antibodies. Hatched oocysts in cyan (1E3-AlexaFluor-647), HA in magenta (anti-HA-AlexaFluor-546), mNeon in green (epifluorescence). B) COWP2-mNeon oocysts hatched and stained only with secondary antibodies AlexaFluor-546 and AlexaFluor-647. C) COWP2-HA oocysts hatched and stained with primary and secondary antibodies. D) COWP2-HA oocysts hatched and stained only with secondary antibodies AlexaFluor-546 and AlexaFluor-647. Images collected on a Zeiss LSM880 Airyscan microscope, confocal mode. Scale bar for all images is 10 µm. Contrast adjustments are the same for all images which are a representative single z-plane. Image collection parameters are the same for each image exposure time 0.01 msec and laser settings: Alexa-647 laser 0.5%; Alexa-546 laser 2%; mNeonGreen laser 2.3%.
(TIF)

S9 Fig *COWP6* and *COWP8* are not expressed in asexual stages or microgamonts. Fluorescence microscopy of HCT-8 cells co-cultured individually with transgenic strains for (A) 12 hours or (B) 48 hours and fixed and processed for imaging. Single nuclei (DAPI, cyan) and staining with 1B5 (asexual marker, Sibley Lab Washington University, yellow or magenta as indicated) indicate trophozoite life cycle stage. Eight nuclei (DAPI, cyan) and staining with 1A5 (asexual marker, Sibley Lab Washington University, yellow or magenta as indicated) indicate meront life cycle stage. Images collected on a widefield epifluorescence microscope; representative images shown. C) Mice were culled at peak infection (faecal NLuc RLU/mg > 500,000) and processed for histology and immunofluorescence. Sixteen nuclei (DAPI, cyan), bullet shape and pattern as previously described used to categorize male parasites [54]. Super resolution images collected on a Zeiss LSM880 Airyscan microscope, airyscan mode.
(TIF)

S10 Fig *COWP8* is not required for resistance to bleach and are still inactivated by heat. A) Experimental design for testing oocyst resistance to bleach and temperature treatment. B) COWP8-mNeon (black) and *∆cowp8* strains (white) remain infectious after treatment with 8% bleach (squares). Average and SD of three technical replicates of one biological replicate. C) COWP8-mNeon (black) and *∆cowp8* strains (white) are equally sensitive to heat inactivation (squares). Average and SD of three technical replicates of biological replicates as indicated.
(TIF)

S11 Fig There is variability in oocyst wall width between replicates. To assess whether this phenotypic difference was consistent between replicates, mean wall width values were separated by replicate and by genotype. There was a significant difference in thickness between WT and *Δcowp8* oocyst walls in the first repeat, but also a significant difference between the width of *Δcowp8* oocysts between replicate one and two. Error bars, IQR; *p < 0.05; ****p *<* 0.0001 (Kruskal-Wallace multiple comparisons test). Raw data reported in S6 Table.
(TIF)

S12 Fig Loss of *COWP8* does not change expression of other *COWP*s. Quantitative proteomics of wild type vs *∆cowp8* plotted as fold change (logFC) and false discovery rate (FDR). Protein extracted from excysted *Cryptosporidium* oocysts and samples labelled with tandem mass tags. *COWP*s indicated in blue, *∆cowp8* reporter proteins in purple. 2 biological replicates, each with 2 technical replicates. See S7 Table for protein identities; upload table to https://plothub.pages.dev/ for interactive visualisation. In our study, we observed the detection of *COWP8* in knockout TMT samples, where it was supposed to be absent. This unexpected detection can be attributed to reporter ion interference and co-isolation [55], which are known limitations of TMT-based quantification in complex samples. Despite this challenge, we opted for TMT labelling due to its significant advantages for our specific research needs. Our in-house proteomic facility consistently achieved superior proteome coverage using TMT compared to label-free approaches. Furthermore, TMT offers a robust method for normalizing batch effects, which is particularly valuable given the labour-intensive nature of *Cryptosporidium* sample acquisition and preparation and the necessity of producing replicates in batches over extended periods. Ultimately, the enhanced proteome coverage, improved normalization capabilities, and increased experimental flexibility provided by TMT were deemed essential for the success this and future multi-batch proteomic study of *Cryptosporidium*, justifying our choice of this methodology.
(TIF)

S13 Fig Force displacement curves for NanoIndentation of oocysts. A) Raw force vs displacement curves from nanoindentation experiments of wild type and *∆cowp8* oocysts. B) Force indentation plot containing all values (insert from A) in range of 0–100 nm of indentation. Three technical replicates performed each for wild type (red, purple, and lilac) and *∆cowp8* (blue, orange, and green). Included in the graph is initial replicate for wild type oocysts (“WT 1”). These values are determined to be outliers. This was the first sample generated during the first few measurements with the machine, when the techniques were being established. When the indentation graph is fitted to the Hertz Model to produce Young’s Modulus data, the outliers range is within that of polystyrene of the dish. Therefore, this sample represents the strength of the dish rather than wild type oocysts. Further optimisation was performed correcting the protocol. WT1 was therefore removed and revised plot of all replicates (excluding wild type biological replicate 1, WT1) are plotted in Fig 5B.
(TIF)

S14 Fig COWP3–5 cannot be endogenously C-terminally tagged. A) Strategy to target the C-terminus of *COWP3* (cgd4_670) for fusion with the full mNeon-3 × HA Repair Cassette, or the simplified 3 × HA tag alone. Both strains include NanoLuciferase-Neomycin resistance fusion protein (NLuc-Neo^R^) expressed by the constitutive Cp*Enolase* promoter. gRNA (black arrow) and regions of 50 bp of homology (grey). Similar strategies designed for B) *cowp4* (cgd8_3350) and C) *cowp5* (cgd7_5150). Neighbouring gene very near the C-terminus of each *COWP* is illustrated in black. D-F) Attempts to generate these mutants was unsuccessful as measured by fecal NLuc from infected mice; limit of detection at 500 RLU/mg, dotted line. Average and SD of three technical replicates of one biological replicate. D) *COWP3* tagging was attempted twice with mNeon Repair cassette and once with simplified 3 × HA tag. E) *COWP4* tagging was attempted once with simplified 3 × HA tag. F) *COWP5* tagging was attempted twice with mNeon Repair cassette and once with simplified 3 × HA tag.
(TIF)

S1 Table Oligonucleotides used in this study. (XLSX)

S2 Table Plasmids created for this study (click name for link). (XLSX)

S3 Table Antibodies and dyes used in this study. (XLSX)

S4 Table Quantitation of Immunoelectron Microscopy Images. Number of images collected and the corresponding number and localisation of gold particles.
(XLSX)

S5 Table Scanning Electron Microscopy scoring data to quantify oocyst phenotype as “smooth” or “wrinkly”. (XLSX)

S6 Table Transmission Electron Microscopy raw data to measure oocyst wall width. (XLSX)

S7 Table Differential Expression Analysis of *C. parvum* Proteins. The table (attached as csv file) presents a summary of the differential expression analysis results. Each row corresponds to a unique gene characterized by the following attributes: “Gene_acc”: a unique integer identifier. “Gene_id”: The gene identifier from CryptoDB. “logFC”: The logarithm (base 2) of the fold change in TMT intensity between the WT (wild type) and KO (*∆cowp8*) samples as computed by the limma R package. “log_AveExpr”: The logarithm (base 2) of the average gene expression level across all samples as computed by the limma R package. “FDR”: The False Discovery Rate-adjusted p-value. Desc: The gene description from CryptoDB. “WT_1” to “WT_4”: The MaxQuant quantified TMT intensity levels for each of wild-type replicates, normalized and fed to the limma package. “KO_1” to “KO_4”: TheMaxQuant quantified TMT intensity levels for each of the overexpression condition, normalized and fed to the limma R package. For enhanced visualization and exploratory data analysis, the table has been formatted to be compatible with the interactive plotting tool available at https://plothub.pages.dev/. This online resource provides an intuitive graphical representation of the data, allowing for immediate visual assessment and interpretation.
(CSV)
