# Supplementary figures and images for: Cryptosporidium oocyst wall proteins are true components of the oocyst wall and COWP8 is not required for parasite transmission
Source: PLoS Pathog. 2025 Oct 23;21(10):e1013561. doi: 10.1371/journal.ppat.1013561 (PMC12548878; doi:10.1371/journal.ppat.1013561)

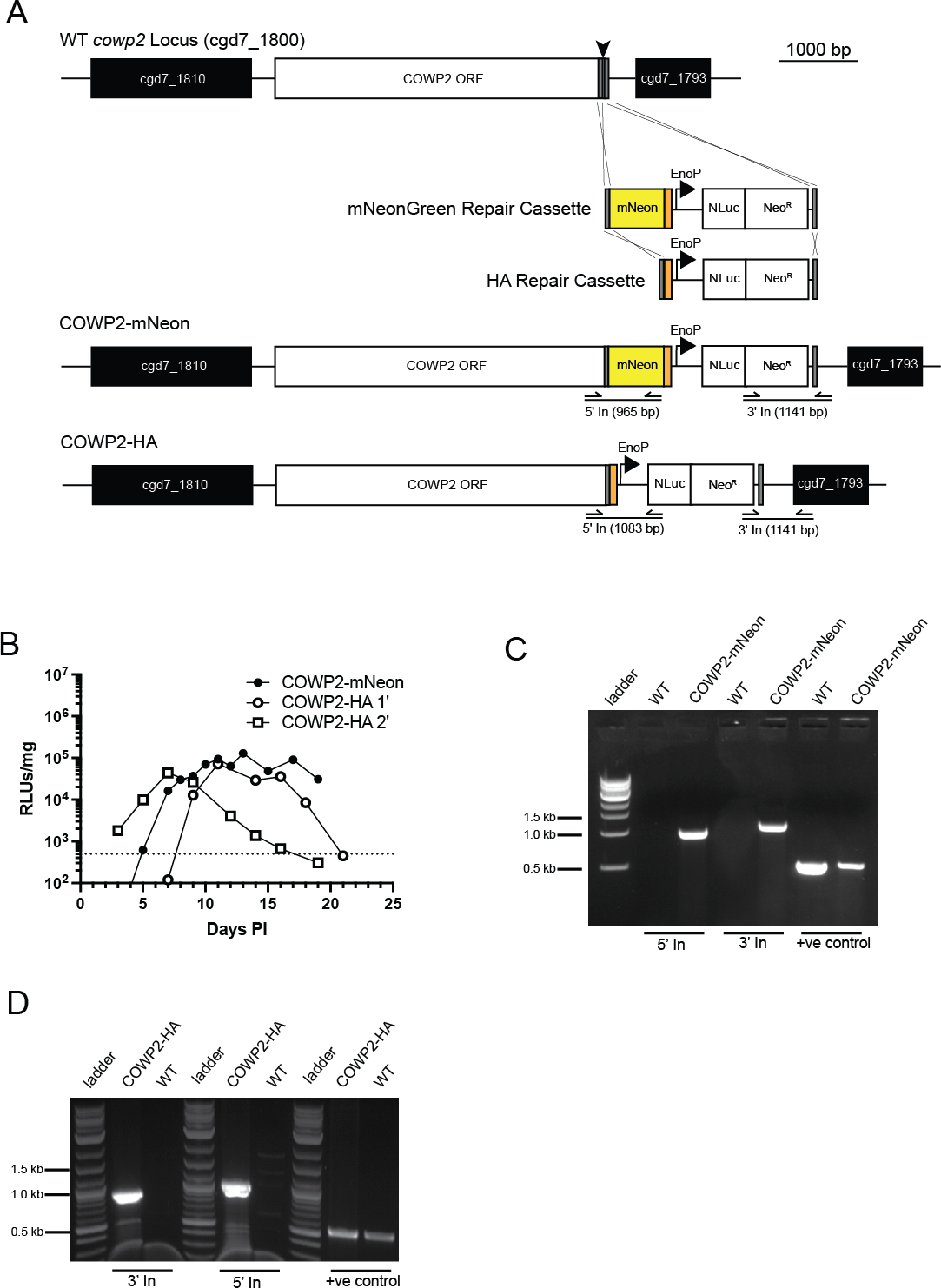

Supplement: S1 Fig — A) gRNA (black arrow) and regions of 50 bp of homology (grey) were used to target the C-terminus of COWP2 (cgd7_1800) for fusion with mNeon (mNeon in yellow and 3 × HA in orange) or the simplified 3 × HA tag alone. Both strains include NanoLuciferase-Neomycin resistance fusion protein (NLuc-NeoR) expressed by the constitutive CpEnolase promoter. B) Infection level of mice as measured by faecal NLuc, limit of detection at 500 RLU/mg, dotted line. Average and SD of three technical replicates of one biological replicate. The first passage of COWP2-mNeon (black circles) was well above the limit of detection. The first passage of COWP2-HA (1’ white circles) and the second passage in mice (2’ white squares) both established robust infections. C-D) PCR with primer pairs indicated in (A) was performed using genomic DNA extracted from wild type and transgenic strains. PCR products confirm correct integration of (C) mNeonGreen or (D) HA repair cassette at the C-terminus of CpCOWP2. (TIF) [file ppat.1013561.s001.tif]

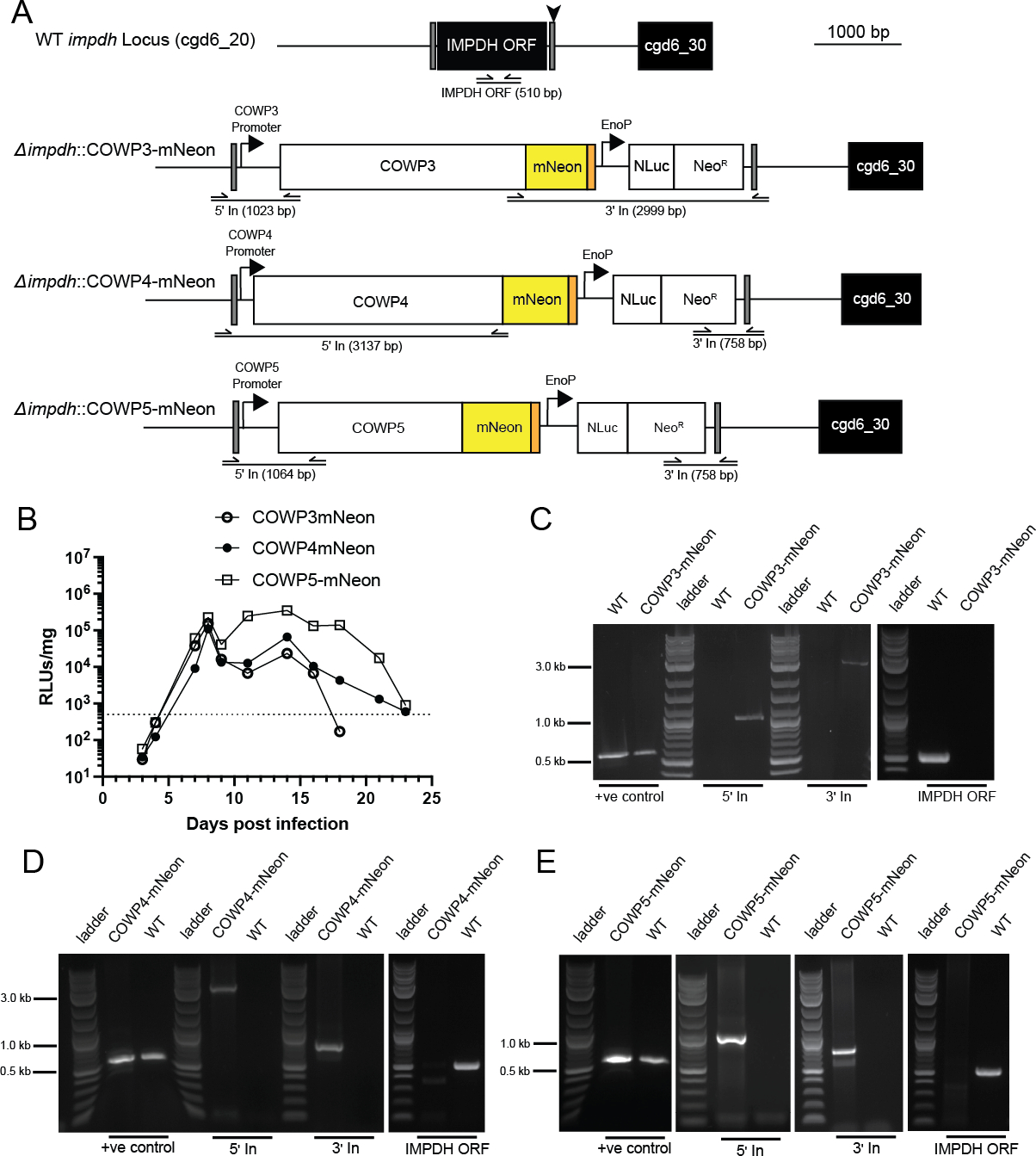

Supplement: S2 Fig — A) Promoter and full open reading frame (ORF) of COWP3, COWP4 and COWP5 each cloned with C-terminal mNeonGreen to generate COWP3/4/5-mNeon repair cassette. Each repair cassette was targeted for integration at Cpimpdh locus (cgd6_20) using gRNA (black arrow) and regions of 50 bp of homology (grey). B) Infection level of mice as measured by faecal NLuc, limit of detection at 500 RLU/mg, dotted line. Average and SD of three technical replicates of one biological replicate. The first passage of ∆impdh::COWP3-mNeon (black circles), ∆impdh::COWP4-mNeon (white circles) and ∆impdh::COWP5-mNeon (white squares) were all well above the limit of detection. C-E) PCR with primer pairs indicated in (A) was performed using genomic DNA extracted from wild type and ∆impdh::COWP3-mNeon (C) ∆impdh::COWP4-mNeon and (D) ∆impdh::COWP5-mNeon (E). (TIF) [file ppat.1013561.s002.tif]

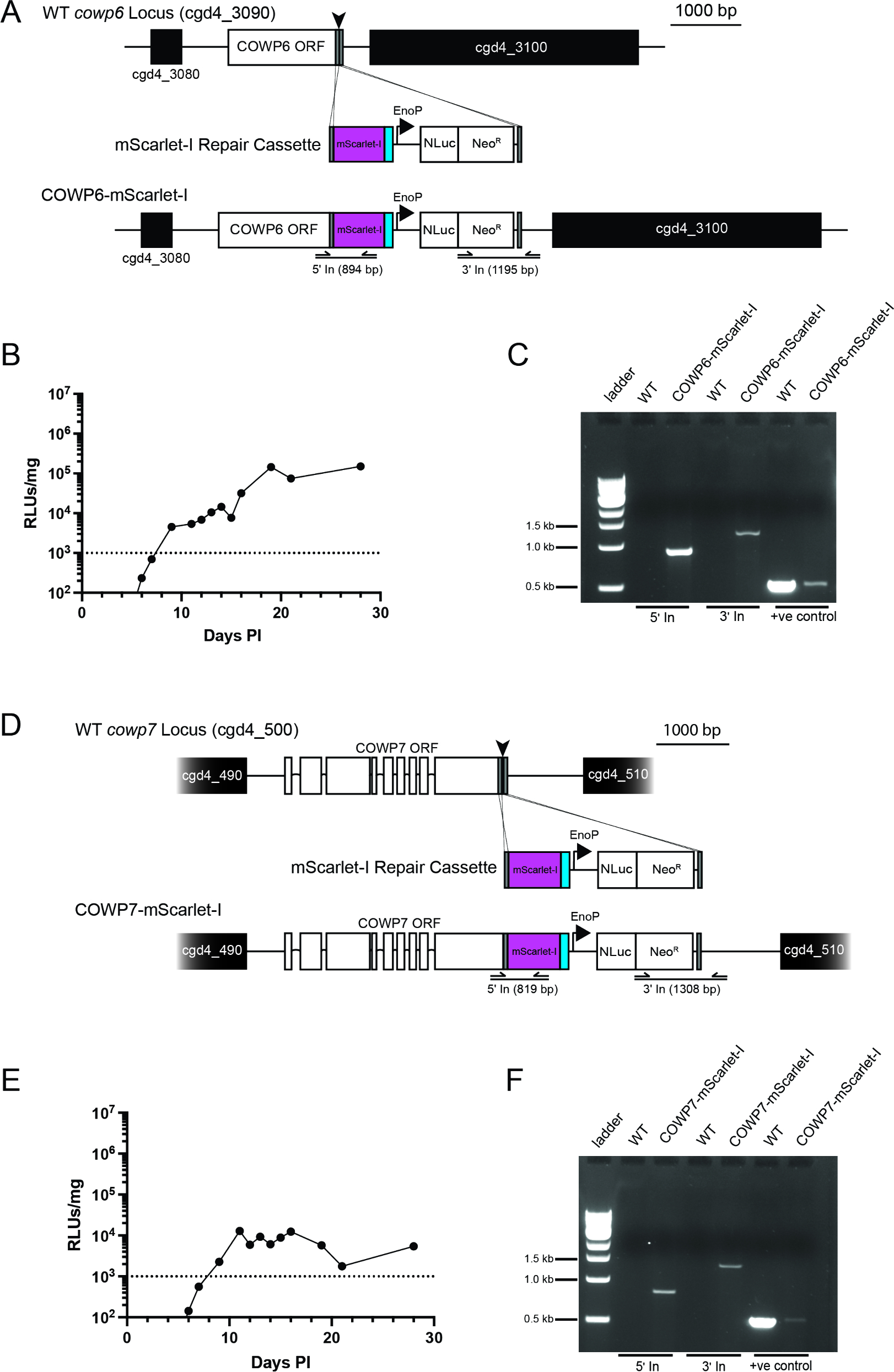

Supplement: S3 Fig — A) Strategy to target the C-terminus of COWP6 (cgd4_3090) for fusion with the mScarlet-I-3 × myc Repair Cassette (mScarlet-I in magenta and 3 × myc in blue). NanoLuciferase-Neomycin resistance fusion protein (NLuc-NeoR) expressed by the constitutive CpEnolase promoter. gRNA (black arrow) and regions of 50 bp of homology (grey). B) Infection level of mice as measured by faecal NLuc, limit of detection at 500 RLU/mg, dotted line. Average and SD of three technical replicates of one biological replicate. The first passage of COWP6-mScarlet-I (black circles) was well above the limit of detection. C) PCR with primer pairs indicated in (A) was performed using genomic DNA extracted from wild type and COWP6-mScarlet-I. D) Strategy to target the C-terminus of COWP7 (cgd4_500) for fusion with the mScarlet-I-3 × myc Repair Cassette (same as reported in A). Cowp7 is predicted to contain 8 introns; exons indicated by white boxes. NanoLuciferase-Neomycin resistance fusion protein (NLuc-NeoR) expressed by the constitutive CpEnolase promoter. gRNA (black arrow) and regions of 50 bp of homology (grey). E) Infection level of mice as measured by faecal NLuc, limit of detection at 500 RLU/mg, dotted line. Average and SD of three technical replicates of one biological replicate. The first passage of COWP7-mScarlet-I (black circles) was well above the limit of detection. F) PCR with primer pairs indicated in (D) was performed using genomic DNA extracted from wild type and COWP7-mScarlet-I. (TIF) [file ppat.1013561.s003.tif]

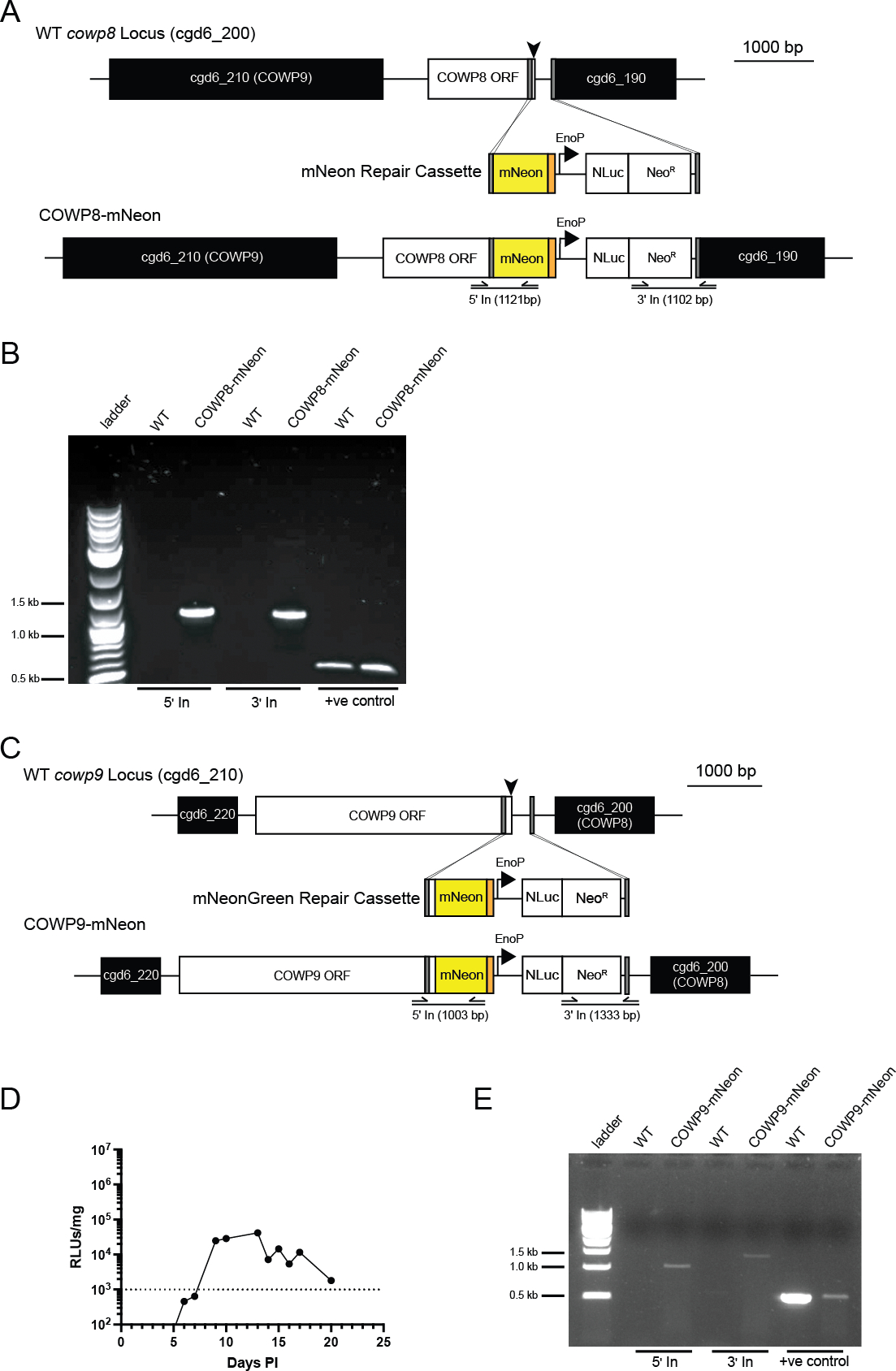

Supplement: S4 Fig — A) Strategy to target the C-terminus of COWP8 (cgd6_200) for fusion with the mNeon-3xHA Repair Cassette (mNeon in yellow and 3 × HA in orange). NanoLuciferase-Neomycin resistance fusion protein (NLuc-NeoR) expressed by the constitutive CpEnolase promoter. gRNA (black arrow) and regions of 50 bp of homology (grey). Note that neighbouring gene upstream of cowp8 is cowp9, illustrated in black. Mouse infections reported in Fig 3A–F. B) PCR with primer pairs indicated in (A) was performed using genomic DNA extracted from wild type and COWP8-mNeon. C) Strategy to target the C-terminus of COWP9 (cgd6_210) for fusion with the mNeon-3 × HA Repair Cassette (same as in A). gRNA (black arrow) and regions of 50 bp of homology (grey). Note that neighbouring gene downstream of cowp9 is cowp8, illustrated in black. D) Infection level of mice as measured by faecal NLuc, limit of detection at 500 RLU/mg, dotted line. Average and SD of three technical replicates of one biological replicate. The first passage of COWP9-mScarlet-I (black circles) was well above the limit of detection. E) PCR with primer pairs indicated in (C) was performed using genomic DNA extracted from wild type and COWP9-mNeon. (TIF) [file ppat.1013561.s004.tif]

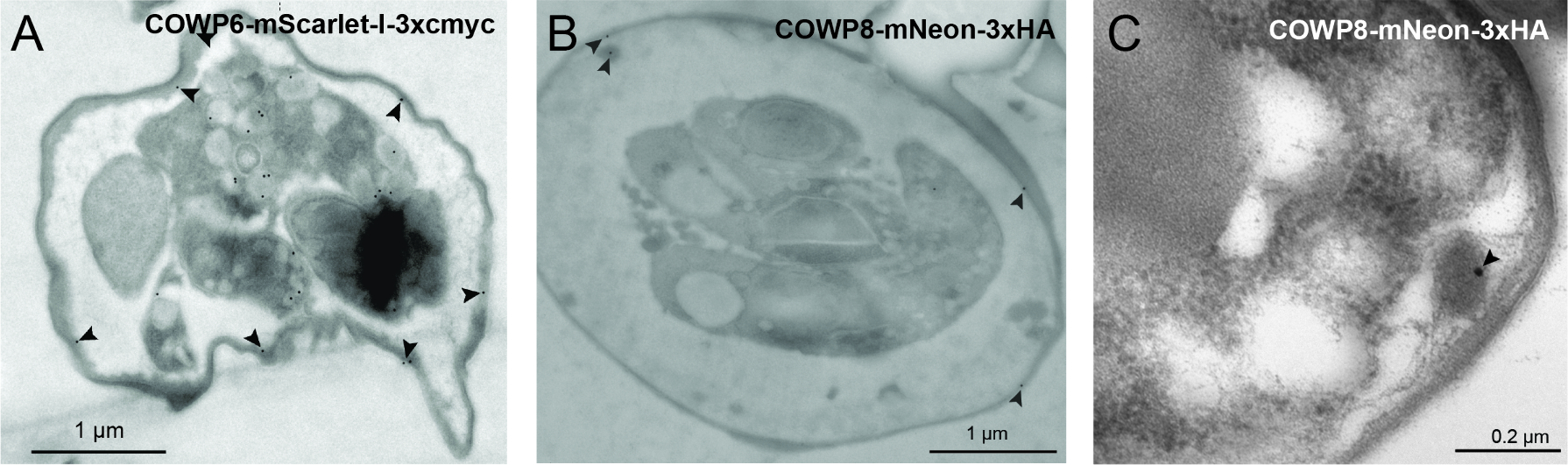

Supplement: S5 Fig — A) Immunoelectron microscopy of COWP6-mScarlet-I confirm localization to the inner layer of the oocyst wall. Representative image shown. B) Immunoelectron microscopy of COWP8-mNeon confirm localization to the inner layer of the oocyst wall. Representative image shown. C) A second localisation in globular-type structures (globule does not appear to be membrane bound) in the space between the sporozoite membrane and inside of the oocyst wall was observed to be positive for COWP8 localisation by Immunoelectron microscopy. Representative image shown. Quantitation provided in S4 Table. (TIF) [file ppat.1013561.s005.tif]

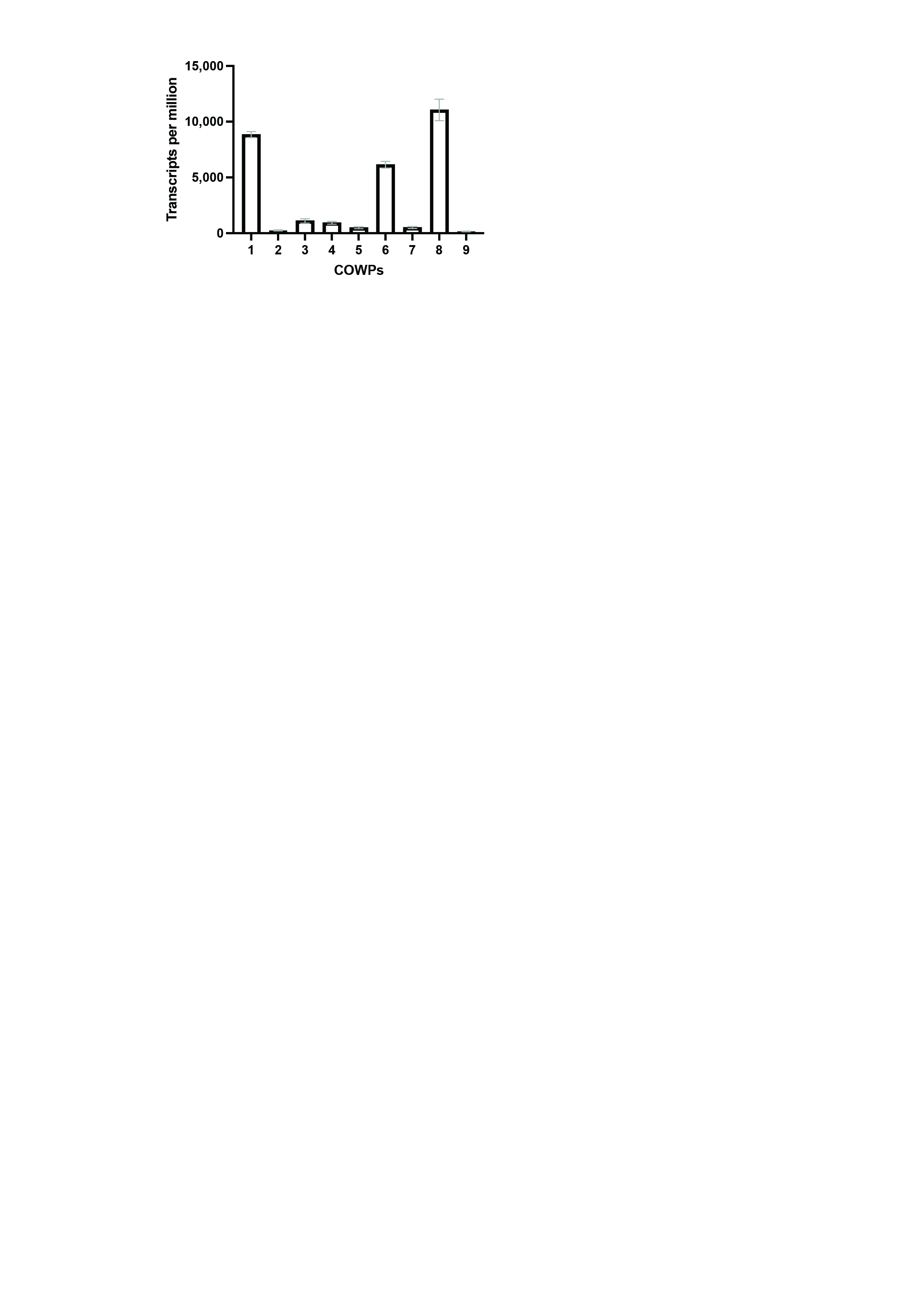

Supplement: S6 Fig — Transcript level of members of the cowp family from “female in vivo” sample from [23] as published on CryptoDB.org [53]. (TIF) [file ppat.1013561.s006.tif]

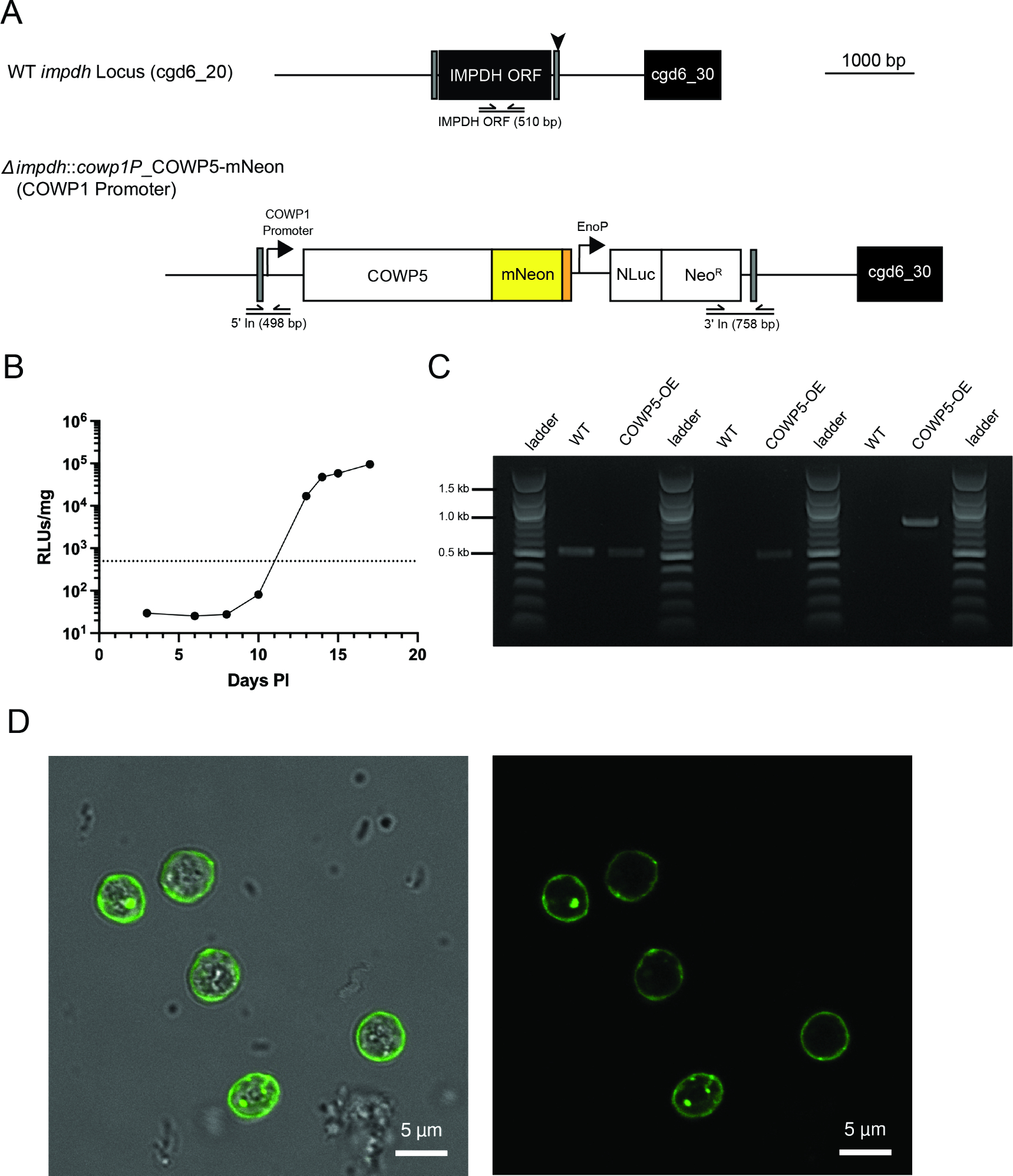

Supplement: S7 Fig — A) The Promoter of cowp1 [23] and full open reading frame (ORF) of cowp5 was cloned with C-terminal mNeonGreen to generate COWP5-mNeon repair cassette. Each repair cassette was targeted for integration at Cpimpdh locus (cgd6_20) using gRNA (black arrow) and regions of 50 bp of homology (grey). B) Infection level of mice as measured by faecal NLuc, limit of detection at 500 RLU/mg, dotted line. Average and SD of three technical replicates of one biological replicate. C) PCR with primer pairs indicated in (A) was performed using genomic DNA extracted from wild type and ∆impdh::cowp1P_COWP5-mNeon. D) ∆impdh::COWP5-mNeon fluorescence microscopy of live oocysts confirms that COWP5 localises to the oocyst wall. Confocal, single z-plane, brightfield: exposure time 0.18 msec, laser power 0.85%. mNeon: exposure time 0.18 msec, laser power 0.85%. (TIF) [file ppat.1013561.s007.tif]

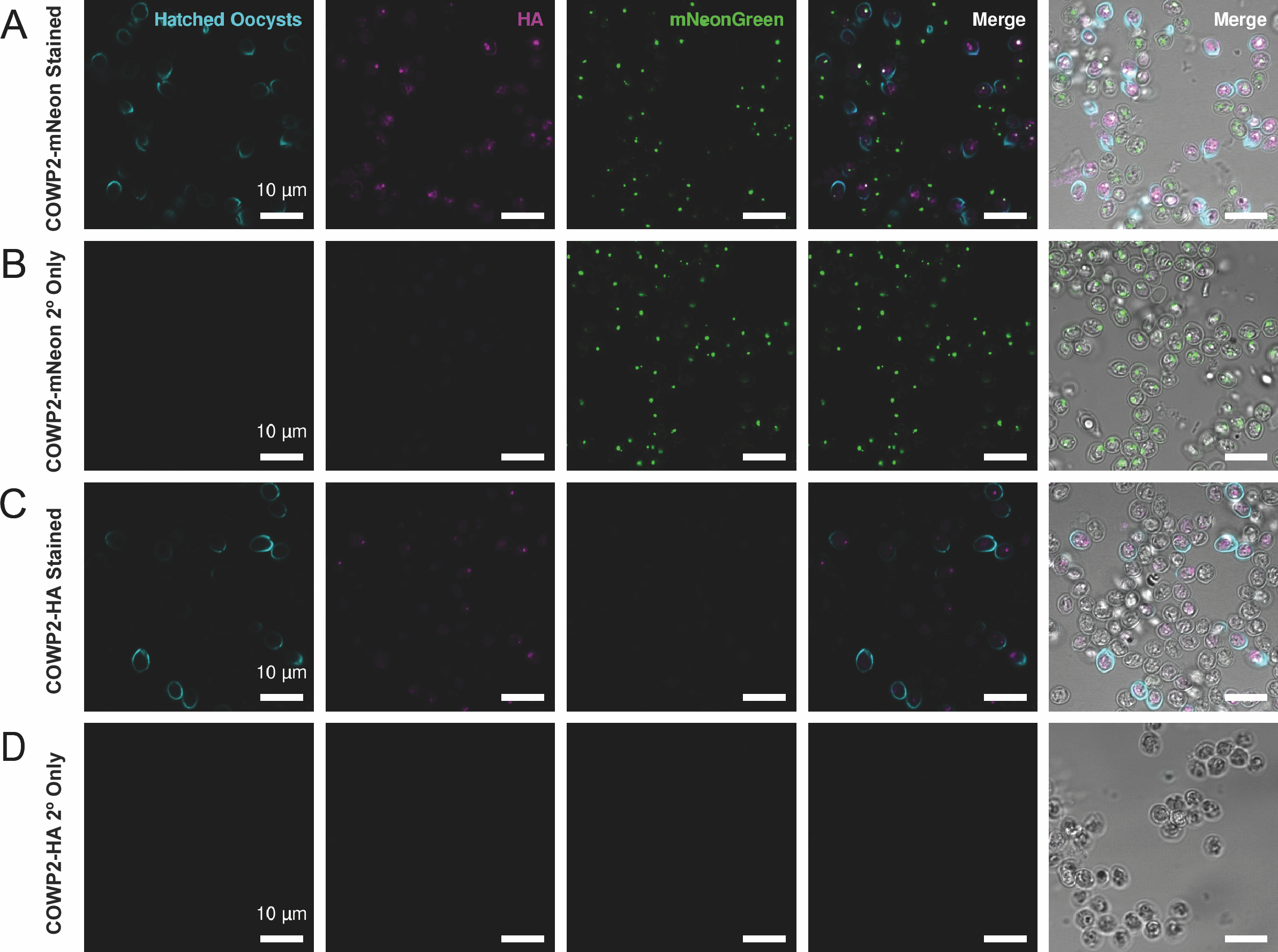

Supplement: S8 Fig — A) COWP2-mNeon oocysts hatched and stained with primary and secondary antibodies. Hatched oocysts in cyan (1E3-AlexaFluor-647), HA in magenta (anti-HA-AlexaFluor-546), mNeon in green (epifluorescence). B) COWP2-mNeon oocysts hatched and stained only with secondary antibodies AlexaFluor-546 and AlexaFluor-647. C) COWP2-HA oocysts hatched and stained with primary and secondary antibodies. D) COWP2-HA oocysts hatched and stained only with secondary antibodies AlexaFluor-546 and AlexaFluor-647. Images collected on a Zeiss LSM880 Airyscan microscope, confocal mode. Scale bar for all images is 10 µm. Contrast adjustments are the same for all images which are a representative single z-plane. Image collection parameters are the same for each image exposure time 0.01 msec and laser settings: Alexa-647 laser 0.5%; Alexa-546 laser 2%; mNeonGreen laser 2.3%. (TIF) [file ppat.1013561.s008.tif]

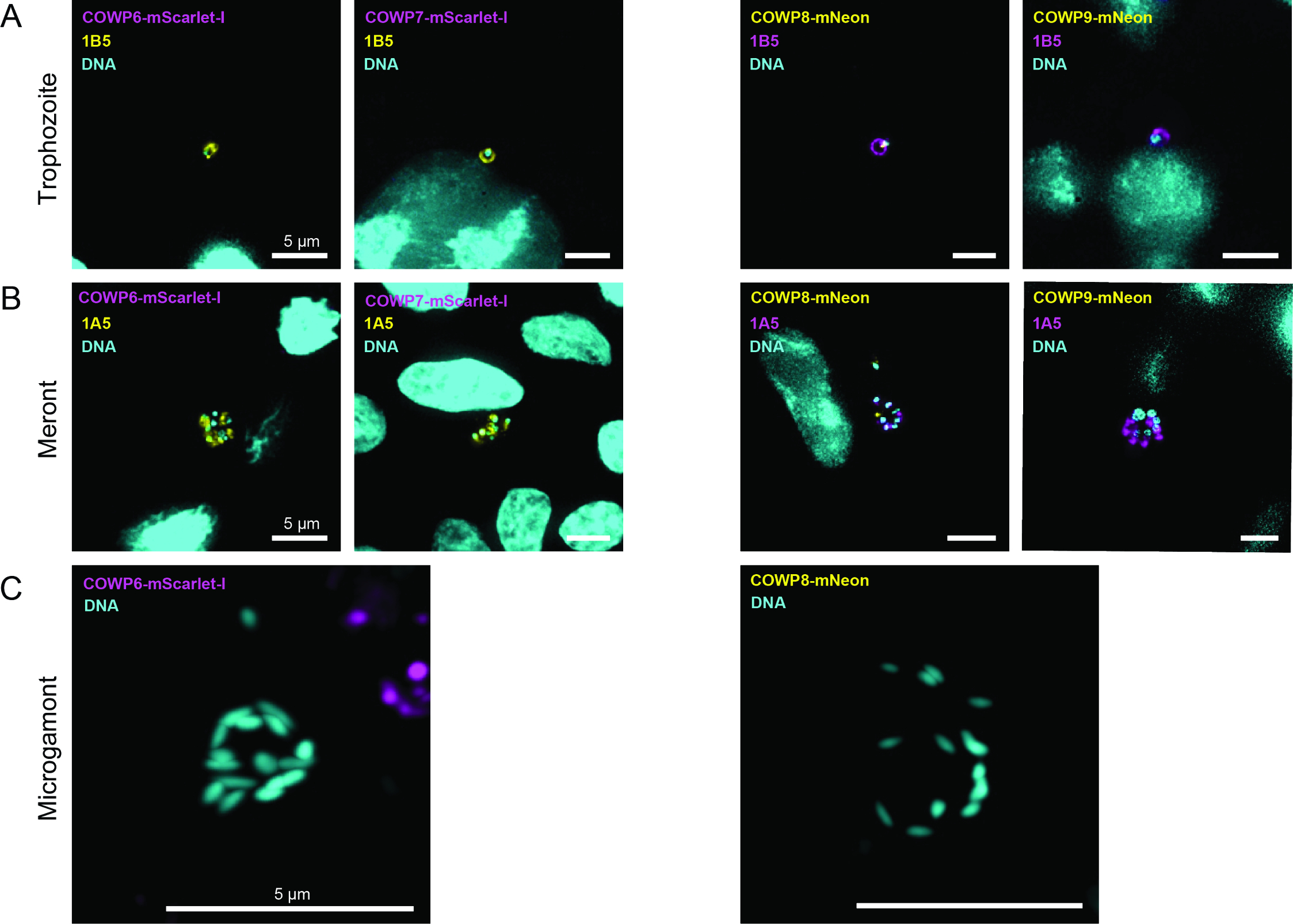

Supplement: S9 Fig — Fluorescence microscopy of HCT-8 cells co-cultured individually with transgenic strains for (A) 12 hours or (B) 48 hours and fixed and processed for imaging. Single nuclei (DAPI, cyan) and staining with 1B5 (asexual marker, Sibley Lab Washington University, yellow or magenta as indicated) indicate trophozoite life cycle stage. Eight nuclei (DAPI, cyan) and staining with 1A5 (asexual marker, Sibley Lab Washington University, yellow or magenta as indicated) indicate meront life cycle stage. Images collected on a widefield epifluorescence microscope; representative images shown. C) Mice were culled at peak infection (faecal NLuc RLU/mg > 500,000) and processed for histology and immunofluorescence. Sixteen nuclei (DAPI, cyan), bullet shape and pattern as previously described used to categorize male parasites [54]. Super resolution images collected on a Zeiss LSM880 Airyscan microscope, airyscan mode. (TIF) [file ppat.1013561.s009.tif]

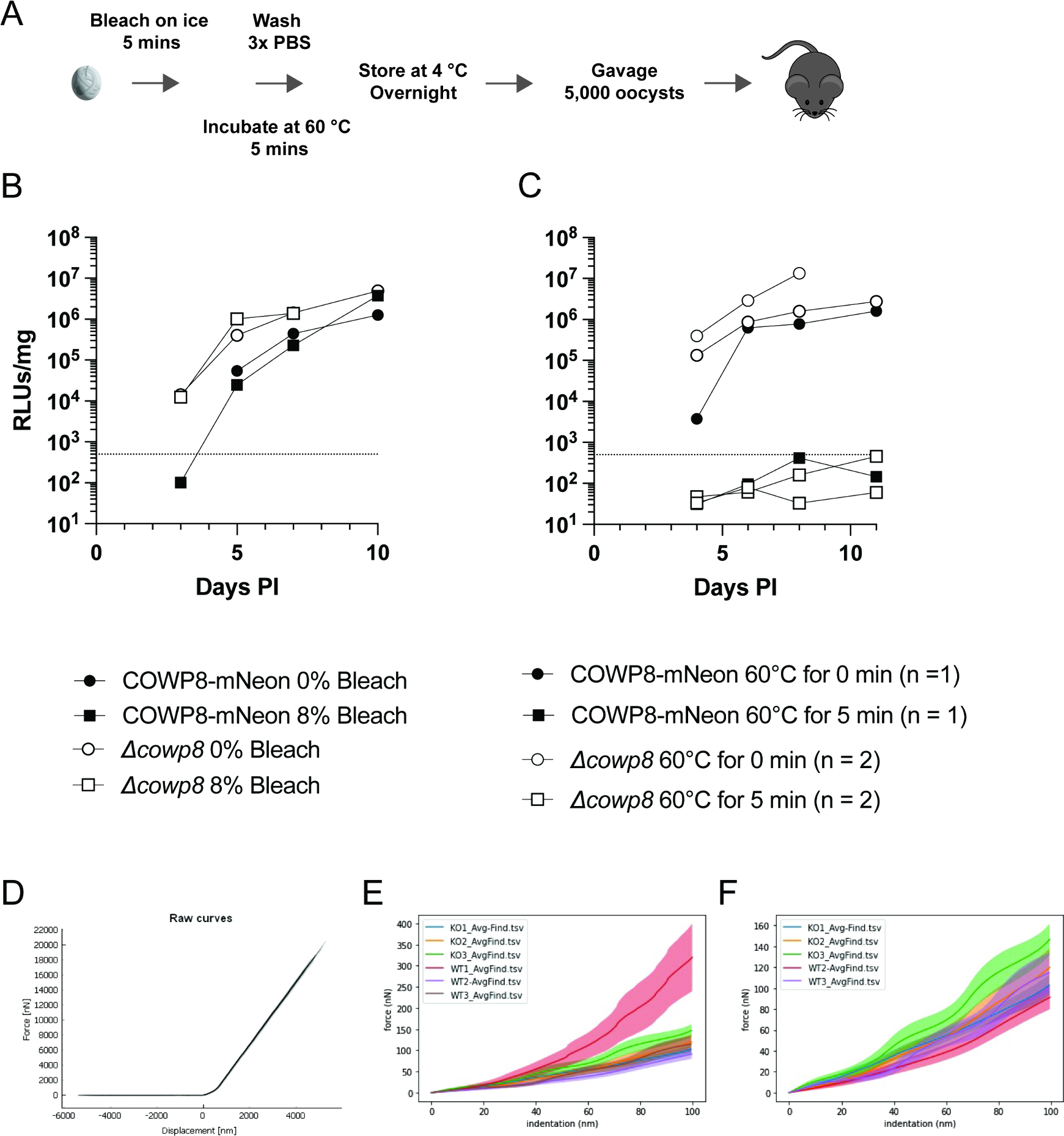

Supplement: S10 Fig — A) Experimental design for testing oocyst resistance to bleach and temperature treatment. B) COWP8-mNeon (black) and ∆cowp8 strains (white) remain infectious after treatment with 8% bleach (squares). Average and SD of three technical replicates of one biological replicate. C) COWP8-mNeon (black) and ∆cowp8 strains (white) are equally sensitive to heat inactivation (squares). Average and SD of three technical replicates of biological replicates as indicated. (TIF) [file ppat.1013561.s010.tif]

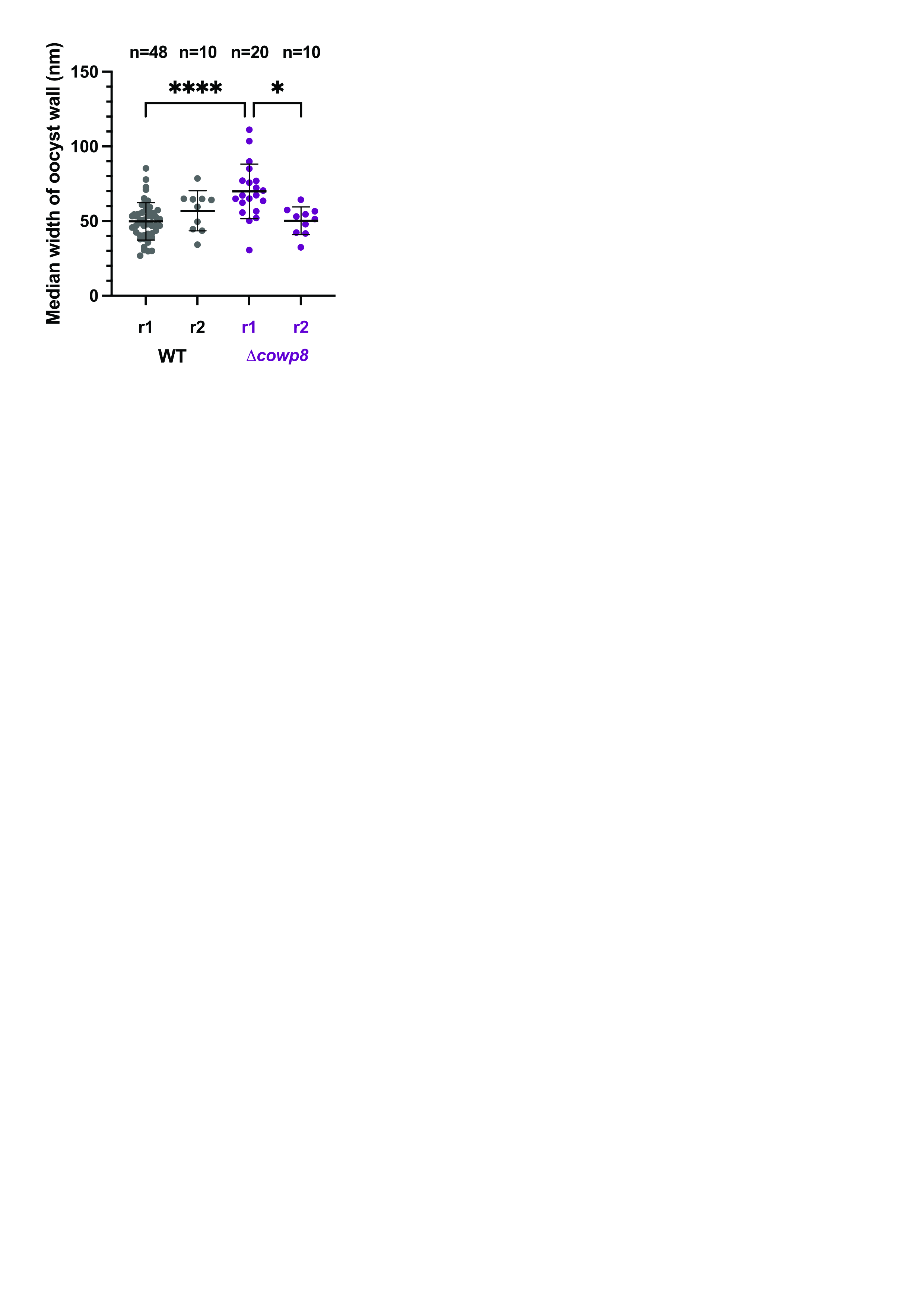

Supplement: S11 Fig — To assess whether this phenotypic difference was consistent between replicates, mean wall width values were separated by replicate and by genotype. There was a significant difference in thickness between WT and Δcowp8 oocyst walls in the first repeat, but also a significant difference between the width of Δcowp8 oocysts between replicate one and two. Error bars, IQR; *p < 0.05; ****p < 0.0001 (Kruskal-Wallace multiple comparisons test). Raw data reported in S6 Table. (TIF) [file ppat.1013561.s011.tif]

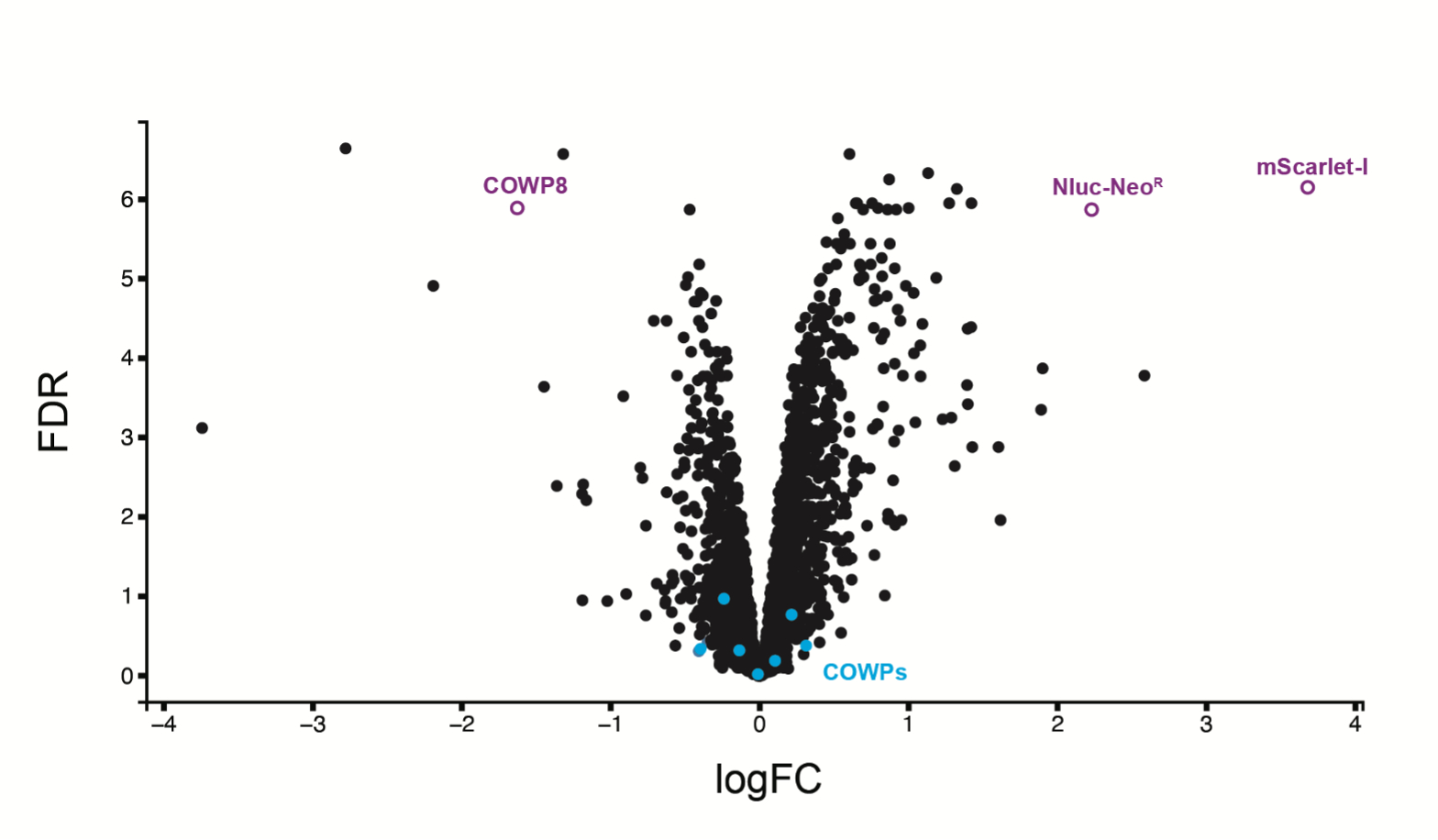

Supplement: S12 Fig — Quantitative proteomics of wild type vs ∆cowp8 plotted as fold change (logFC) and false discovery rate (FDR). Protein extracted from excysted Cryptosporidium oocysts and samples labelled with tandem mass tags. COWPs indicated in blue, ∆cowp8 reporter proteins in purple. 2 biological replicates, each with 2 technical replicates. See S7 Table for protein identities; upload table to https://plothub.pages.dev/ for interactive visualisation. In our study, we observed the detection of COWP8 in knockout TMT samples, where it was supposed to be absent. This unexpected detection can be attributed to reporter ion interference and co-isolation [55], which are known limitations of TMT-based quantification in complex samples. Despite this challenge, we opted for TMT labelling due to its significant advantages for our specific research needs. Our in-house proteomic facility consistently achieved superior proteome coverage using TMT compared to label-free approaches. Furthermore, TMT offers a robust method for normalizing batch effects, which is particularly valuable given the labour-intensive nature of Cryptosporidium sample acquisition and preparation and the necessity of producing replicates in batches over extended periods. Ultimately, the enhanced proteome coverage, improved normalization capabilities, and increased experimental flexibility provided by TMT were deemed essential for the success this and future multi-batch proteomic study of Cryptosporidium, justifying our choice of this methodology. (TIF) [file ppat.1013561.s012.tif]

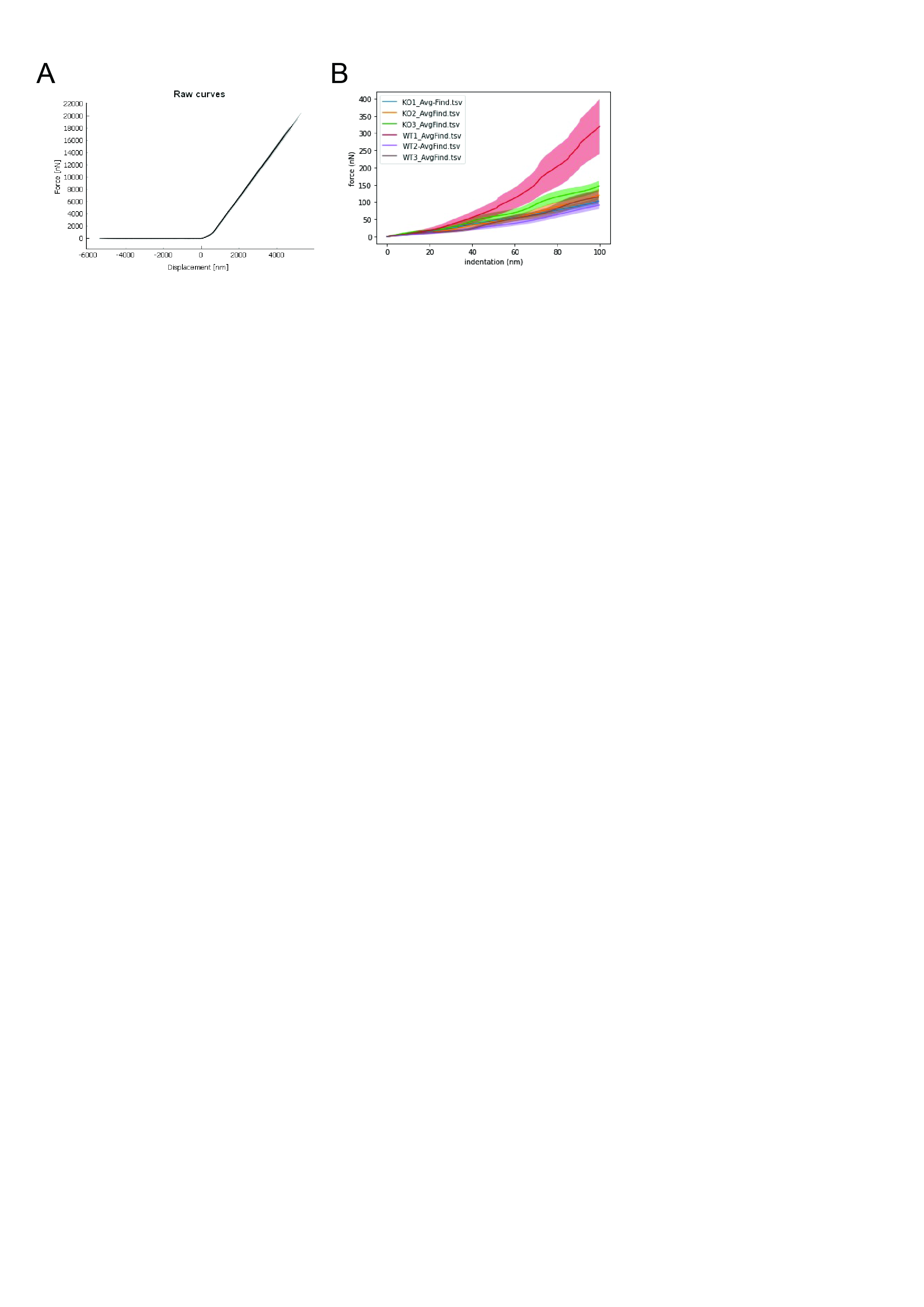

Supplement: S13 Fig — A) Raw force vs displacement curves from nanoindentation experiments of wild type and ∆cowp8 oocysts. B) Force indentation plot containing all values (insert from A) in range of 0–100 nm of indentation. Three technical replicates performed each for wild type (red, purple, and lilac) and ∆cowp8 (blue, orange, and green). Included in the graph is initial replicate for wild type oocysts (“WT 1”). These values are determined to be outliers. This was the first sample generated during the first few measurements with the machine, when the techniques were being established. When the indentation graph is fitted to the Hertz Model to produce Young’s Modulus data, the outliers range is within that of polystyrene of the dish. Therefore, this sample represents the strength of the dish rather than wild type oocysts. Further optimisation was performed correcting the protocol. WT1 was therefore removed and revised plot of all replicates (excluding wild type biological replicate 1, WT1) are plotted in Fig 5B. (TIF) [file ppat.1013561.s013.tif]

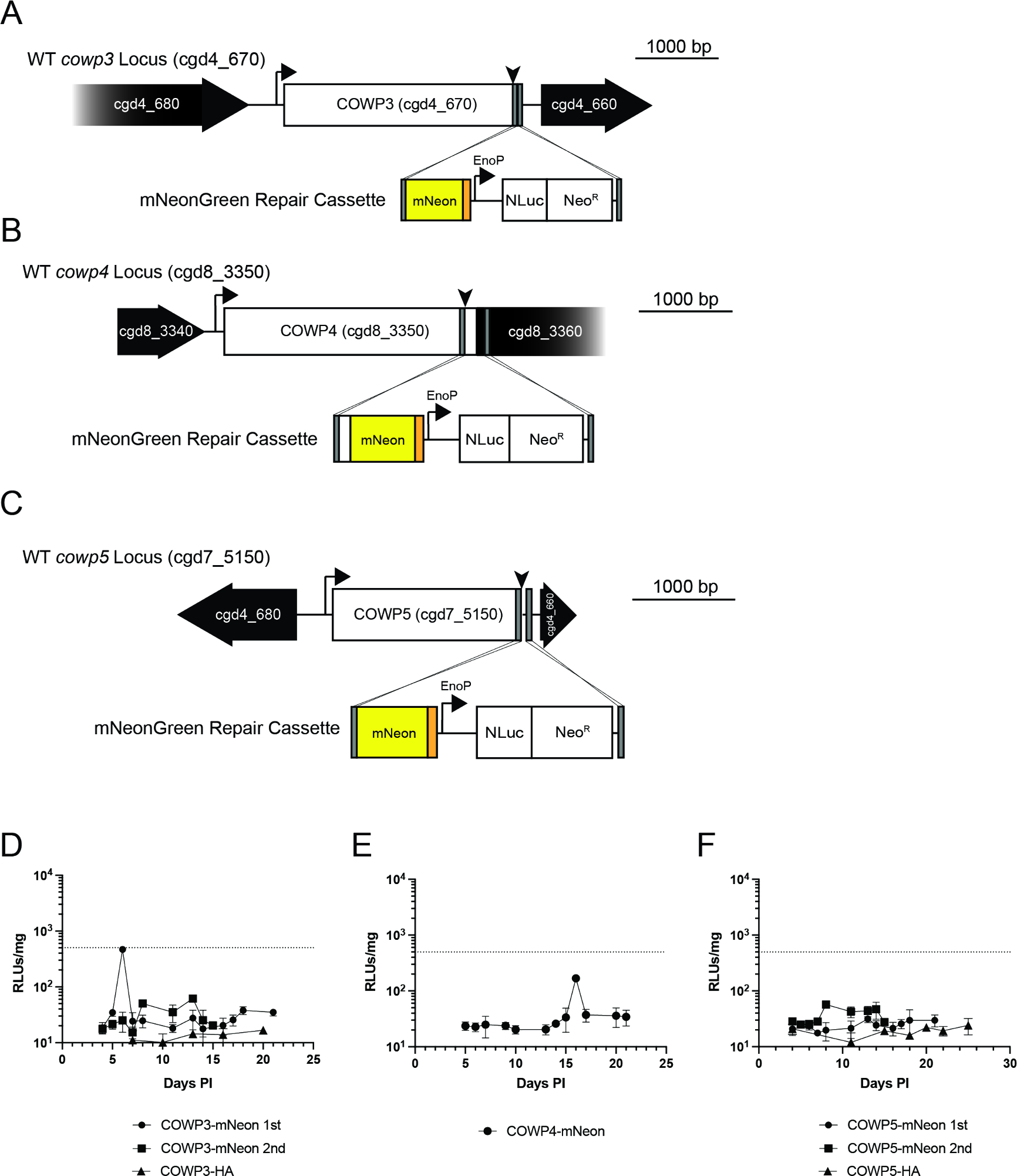

Supplement: S14 Fig — A) Strategy to target the C-terminus of COWP3 (cgd4_670) for fusion with the full mNeon-3 × HA Repair Cassette, or the simplified 3 × HA tag alone. Both strains include NanoLuciferase-Neomycin resistance fusion protein (NLuc-NeoR) expressed by the constitutive CpEnolase promoter. gRNA (black arrow) and regions of 50 bp of homology (grey). Similar strategies designed for B) cowp4 (cgd8_3350) and C) cowp5 (cgd7_5150). Neighbouring gene very near the C-terminus of each COWP is illustrated in black. D-F) Attempts to generate these mutants was unsuccessful as measured by fecal NLuc from infected mice; limit of detection at 500 RLU/mg, dotted line. Average and SD of three technical replicates of one biological replicate. D) COWP3 tagging was attempted twice with mNeon Repair cassette and once with simplified 3 × HA tag. E) COWP4 tagging was attempted once with simplified 3 × HA tag. F) COWP5 tagging was attempted twice with mNeon Repair cassette and once with simplified 3 × HA tag. (TIF) [file ppat.1013561.s014.tif]
